# Out of the Qinghai–Tibetan Plateau and rapid radiation across Eurasia for *Allium* section *Daghestanica* (Amaryllidaceae)

**DOI:** 10.1093/aobpla/plab017

**Published:** 2021-04-14

**Authors:** Min-Jie Li, Huan-Xi Yu, Xian-Lin Guo, Xing-Jin He

**Affiliations:** 1 Key Laboratory of Bio-Resources and Eco-Environment of Ministry of Education, College of Life Sciences, Sichuan University, Chengdu, Sichuan 610065, P.R. China; 2 State Key Laboratory of Grassland Agro-Ecosystem, Institute of Innovation Ecology & School of Life Science, Lanzhou University, Lanzhou, Gansu 730000, P.R. China; 3 Nanjing Institute of Environmental Science, MEE, Nanjing, Jiangsu 210042, P.R. China

**Keywords:** Ecological differentiation, Eurasian disjunction, evolutionary radiation, section *Daghestanica*

## Abstract

The disjunctive distribution (Europe–Caucasus–Asia) and species diversification across Eurasia for the genus *Allium* sect. *Daghestanica* has fascinating attractions for researchers aiming to understanding the development and history of modern Eurasia flora. However, no any studies have been carried out to address the evolutionary history of this section. Based on the nrITS and cpDNA fragments (trnL–trnF and rpl32–trnL), the evolutionary history of the third evolutionary line (EL3) of the genus *Allium* was reconstructed and we further elucidated the evolutionary line of sect. *Daghestanica* under this background. Our molecular phylogeny recovered two highly supported clades in sect. *Daghestanica*: the Clade I includes Caucasian–European species and Asian *A. maowenense*, *A. xinlongense* and *A. carolinianum* collected in Qinghai; the Clade II comprises Asian yellowish tepal species, *A. chrysanthum*, *A. chrysocephalum*, *A. herderianum*, *A. rude* and *A. xichuanense*. The divergence time estimation and biogeography inference indicated that Asian ancestor located in the Qinghai–Tibetan Plateau (QTP) and the adjacent region could have migrated to Caucasus and Europe distributions around the Late Miocene and resulted in further divergence and speciation; Asian ancestor underwent the rapid radiation in the QTP and the adjacent region most likely due to the heterogeneous ecology of the QTP resulted from the orogeneses around 4–3 million years ago (Mya). Our study provides a picture to understand the origin and species diversification across Eurasia for sect. *Daghestanica*.

## Introduction

*Allium*, the only member of the monotypic tribe Allieae within the subfamily Allioideae (Amaryllidaceae) (APG IV 2016), is naturally distributed throughout the Northern Hemisphere and is represented by ca. 920 species (Govaerts *et al*. 2005–2014). The major centre of diversity and diversification of *Allium* is located in Southwest and Central Asia and the Mediterranean region (Choi and Oh 2011). Molecular phylogenies have demonstrated that *Allium* currently comprises 15 subgenera and 72 sections along three independent evolutionary lines (Friesen *et al.* 2006; Li *et al.* 2010). Recent phylogenetic studies have presented a clear evolutionary history for the former two evolutionary lines (EL1 and EL2) (e.g. Wheeler *et al.* 2013; Herden *et al.* 2016), but a rather complex evolution history for the EL3 due to the prevalence of polymorphisms both in morphology and genetic phylogeny (Li *et al.* 2010; Li *et al.* 2016a; Choi *et al.* 2012; Huang *et al.* 2014; Sinitsyna *et al.* 2016; Xie *et al.* 2019a). The EL3 contains 60 % of *Allium* species mainly distributing in Eurasia and displays high genetic and morphological polymorphisms both among species (Li *et al.* 2016a; Xie *et al.* 2019a) and within species (Huang *et al.* 2014; Li *et al.* 2019). The EL3 comprises subgenera *Butomissa*, *Cyathophora*, *Rhizirideum*, *Allium*, *Cepa*, *Reticulatobulbosa* and *Polyprason*. Of which, Asian endemic subgenera *Butomissa* and *Cyathophora* form the first two basal lineages and are successively sister to the complex clade constitutive of the remaining subgenera (Li *et al.* 2010). In this study, we focused on subg. *Polyprason* sect. *Daghestanica*, which mainly expands throughout Eurasian temperate zone. Towards this situation, it is expected that the evolutionary investigation of this section could provide insights for the development and history of modern Eurasian flora.

The molecular phylogeny of *Allium* revealed that sect*. Daghestanica* species are mainly distributed in three disjunctive regions: Europe (mainly from the eastern Alps to the Pyrenees), Caucasus and Asia [mainly the Qinghai–Tibetan Plateau (QTP) of China] (Friesen *et al.* 2006; Li *et al.* 2010). European group comprises *A. ericetorum*, *A. ochroleucum*, *A. kermesinum* and *A. suaveolens*, showing semi-cylindrical or narrowly linear leaves, bulb tunics splitting into longitudinal stripes (but not into fibres) and per inflorescence with the lower number of flowers; Caucasian group includes *A. gunibicum* and *A. daghestanicum*, having thin thread-like leaves, bulb tunics thinly leathery, splitting, and the densely many flowers per inflorescence in *A. daghestanicum* but the lower number of flowers in *A. gunibicum*; Asian group contains *A. chrysocephalum*, *A. chrysanthum*, *A. herderianum*, *A. maowenense*, *A. rude* and *A. xichuanense* (Li *et al.* 2010), presenting semiterete, terete or linear leaves, bulb tunics thinly leathery, splitting, and densely many flowers per inflorescence (Xu and Kamelin 2000). However, among Asian species, *A. maowenense* exhibits perianth similarity with European species due to the whitish tepals, but a leaf similarity with Caucasian species due to flexuous leaves rather than erect-angled leaves. Recently, different new species of this section have been described (Friesen *et al.* 2020; Xie *et al.* 2020b). The phylogeny of *A. xinlongense* showed this new species has a close genetic relationship with Asian species and is endemic to the QTP. *Allium xinlongense* has whitish tepals, similar to Asian *A. maowenense* and European species, while bulb and densely many flowers with reddish midvein similar to Caucasian *A. daghestanicum* (Xie *et al.* 2020b). Another new species *A. matinae* was found in north-western Iran, which shows a morphological similarity with European group due to the lower number of flowers per inflorescence. The genetic phylogeny showed a genetic affinity between *A. matinae* and Caucasian *A. gunibicum* and *A. daghestanicum* (Friesen *et al.* 2020). Among these three geographical groups, only Caucasian group begins with anthesis in autumn, while European and Asian groups are flowering in summer (Xu and Kamelin 2000; Friesen *et al.* 2006). It is therefore interesting to know the evolutionary history of these three geographical groups, which would provide a picture for people to understand the evolutionary history of sect. *Daghestanica* among the disjunctive areas (Friesen *et al.* 2020).

**Figure 1.**
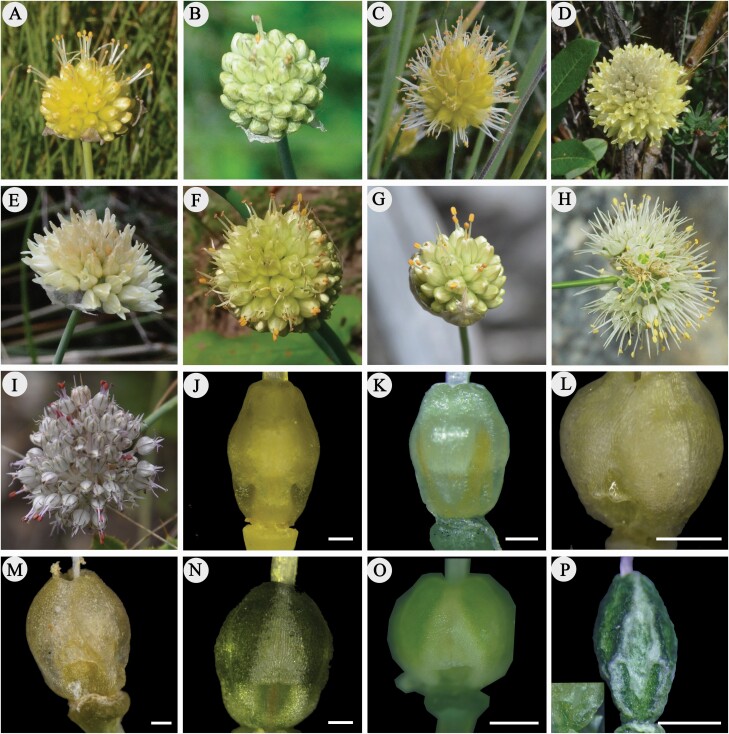
Flower and nectary traits of Asian sect. *Daghestanica* species. (A–B, J) *A. rude*; (C, K) *A. chrysanthum*; (D, L) *A. chrysocephalum*; (E, M) *A. herderianum*; (F–G, N) *A. xichuanense*; (H, O) *A. maowenense*; (I, P) *A. xinlongense*. Scale bar 0.5 mm.

Additional attention should be paid for Asian group due to the larger species number and interspecific morphological polymorphisms (Fig. 1). Asian species mainly are endemic to the QTP and the adjacent region. Phylogenies of six Asian species have been investigated based on the whole-chloroplast genomes and transcriptomes (Xie *et al.* 2019b; Xie *et al.* 2020c). However, these studies just used only one individual for each species and the relationships among the three geographic groups were also not investigated. It has been proven that the recent evolutionary radiations are common in nearly all main lineages of land plants, particularly in angiosperms (e.g. Kay *et al.* 2005; Nagalingum *et al.* 2011; Horn *et al.* 2014; Wang *et al.* 2017), which has led extensively morphological and genetic radiation to the local biodiversity (e.g. Richardson *et al.* 2001; Hughes and Eastwood 2006; Wang *et al.* 2009; Zhang *et al.* 2014; Pirie *et al.* 2016; Mosbrugger *et al.* 2018). Resulting from the continuous interactions between climatic and geological settings (Favre *et al.* 2015; Renner 2016; Silvestro and Schnitzler 2018), the QTP is characterized by a heterogeneous geomorphological and environmental mosaic, and consequently fosters high plant diversity and endemism, especially in the Eastern Himalaya and the Mountains of Southwest China (i.e. Hengduan Mountain Region, HMR) (e.g. Xu *et al.* 2014; Ebersbach *et al.* 2017; Favre *et al.* 2016; Xing and Ree 2017; Mosbrugger *et al.* 2018). The recent radiation lineages are the important components of biodiversity of these regions (e.g. Wen *et al.* 2014; Zhang *et al.* 2016; Ebersbach *et al.* 2017; Sun *et al.* 2017). The more studies of species radiation or rapid episodes of species diversity mean the richer source of new insights into origins of local biodiversity. It therefore expected that recovering the evolutionary radiation of Asian sect. *Daghestanica* species in the QTP can provide more insights for local biodiversity.

In this study, we reconstructed the evolutionary history of the three disjunctive geographical groups of sect. *Daghestanica* under the phylogenetic structure of EL3 based on the whole ITS sequence and cpDNA fragments (trnL–trnF and rpl32–trnL), aiming to infer the phylogeny of sect. *Daghestanica*, and the possibilities of origin and diversification of these species across Eurasia.

## Materials and Methods

### Plant sampling

The identification and sampling of Asian sect. *Daghestanica* species were carried out with field investigations. We collected 52 individuals from 48 populations for DNA sequencing, including one *A. herderianum*, three individuals of *A. xichuanense* from three populations, 21 individuals of *A. rude* from 21 populations, six individuals of *A. chrysocephalum* from six populations, four individuals of *A. chrysanthum* from three populations, 10 individuals of *A. xinlongense* from eight populations, two individuals of *A. maowenense* from one population and five individuals of *A. carolinianum* from five populations **[see****Supporting Information—Table S1****]**. Species discriminations are based on the diagnostic traits provided by the Flora of China (Xu and Kamelin 2000). Fresh leaves collected in the field were immediately placed in the preservation box with silica gel to dry. Voucher specimens of all samples were stored at Sichuan University Herbarium (SZ!).

### Anatomy of leaves

Leaf anatomy was examined using silica-dried leaf blades collected usually before the flowering stage when foliage senescence begins, with one sample for each species. The leaf tissue was fixed in Carnoy (6:3:1, v/v/v, absolute ethanol:chloroform:glacial acetic acid) and embedded in paraffin; the transverse sections (10- to 12-μm thick) were double-stained with safranin and fast green. Afterwards, they were examined using an Olympus DP71 light microscope and photographed using an Olympus BX5 digital camera.

### Macromorphological records on bulbs and flowers

We treated the bulb shape, flower (perianth) colour, perianth midvein, perianth shape, nectary shape and projection shape covering on the nectary as macromorphological traits. We observed these macrotraits at different populations for multiple individuals in the wild investigations to assure the trait stability and recorded their variation. Bulbs and fresh flowers were photographed using a Nikon D90 camera. Nectary was observed and photographed using a Nikon SMZ25 dissecting microscope.

### Microsculpturing of leaves, seeds and pollen grains

Two samples were examined for each species, except *A. rude* for which four or six samples were investigated due to a greater number of samples **[see****Supporting Information—Table S1****]**. The mature seeds were examined under the stereomicroscope. After dehydration via an ethanol series, the samples of leaves, seeds and pollen grains were mounted directly on aluminium stubs using double adhesive tape and were coated with gold-palladium (ca. 25 nm). The microsculpturing of the investigated samples was studied under a JSM-7500F scanning electron microscope (SEM) and were photographed at high magnifications.

### DNA amplification and sequence data analysis

Genomic DNA of each individual was extracted from the dried leaves using a plant genomic DNA kit (Tiangen Biotech, Beijing, China). Three cpDNA fragments, trnL–trnF, rpl32–trnL and rps16, and the entire nrITS, were amplified by polymerase chain reaction (PCR) with the universal primers (White *et al.* 1990; Shaw *et al.* 2005). For nrITS, the cycling conditions began at 94 °C for 4 min, followed by 38 cycles at 94 °C for 1 min, 55 °C for 45 s and 72°C for 1 min with a final extension at 72 °C for 10 min. For trnL–trnF and rpl32–trnL and rps16, the PCR conditions mostly were the same, except the denaturation temperatures, respectively, at 60, 56 and 52 °C. Amplified products were purified and then sequenced using the universal primers. Newly produced sequences were submitted in GenBank with accession number MN866527**–**MN866746**[see****Supporting Information—Table S2****]**.

### Sequence alignment and phylogenetic analyses

To examine the evolutionary history of sect. *Daghestanica*, we downloaded the available nrITS and cpDNA (trnL–trnF, rpl32–trnL and rps16) sequences of the EL3 from GenBank **[see****Supporting Information—Fig. S5**; **Supporting Information—Table S3****]**. Due to the limited rps16 sequences for the EL3, we just concatenated the trnL–trnF and rpl32–trnL sequences as the cpDNA data set. Among these sequences, 15 trnL–trnF sequences, 15 rpl32–trnL sequences **[see****Supporting Information—Table S3****]** and 29 nrITS sequences **[see****Supporting Information—Fig. S5****]** were, respectively, retrieved for Caucasian group, European group and some of Asian species in sect. *Daghestanica***[see****Supporting Information—Fig. S5**; **Supporting Information—Table S3****]**. We used SeqMan (DNAstar; Burland 2000) to edit the newly produced DNA sequences and obtain consensus sequences. The sequences were aligned using MEGA 7 (Kumar *et al.* 2016) prior to the manual adjustment. Indels were treated as missing data during the phylogenetic analyses.

Bayesian inference (BI) analyses were carried out using MrBayes version 3.2 (Ronquist *et al.* 2012). Based on the Akaike information criterion (AIC) implemented in MrModelTest 2.2 (Nylander 2004), the best-fitting nucleotide substitution model (GTR+I+G) was inferred for each data set. We ran Bayesian approach from a random starting tree using a Markov Chain Monte Carlo (MCMC) analysis with three heated chains (0.1 temperature increments) and one cold chain, and employing 20 000 000 generations for all analyses. Two independent runs were performed for each analysis, and trees were drawn every 1000 generations. The first 20 % trees were discarded as burn-in and the post-burn trees were used to construct the majority-consensus trees, and to estimate the posterior probabilities. The maximum likelihood (ML) trees were reconstructed in Standard RAxML (Stamatakis 2014), with 100 bootstrap replicates to find the best-scoring ML tree.

To further validate the evolutionary relationships of sect. *Daghestanica*, we used SplitsTree4 (Hudson and Bryant 2006) to reconstruct phylogenetic neighbour-net graphics just including the samples of sect. *Daghestanica* and *A. carolinianum* 1–4 collected in Qinghai. The analyses were performed based on the nrITS and the combined cpDNA sequences (trnL–trnF and rpl32–trnL) by calculating uncorrected sequence divergence (i.e. uncorrected *P*-distance), with 1000 replicates for bootstrap resampling.

### Divergence time estimation

In the absence of known fossils of *Allium* or its closely related genera, we used secondary calibration approaches (Schenk 2016) by defining a normal prior distribution in BEAST v.2.5.0 (Bouckaert *et al.* 2014) to estimate the divergence time of the EL3 under which the divergence time of sect*. Daghestanica* was further checked. Xie *et al.* (2020a) used the secondary calibration approach to date back the origin time of *Allium* to 41.932 million years ago (Mya) based on the whole-chloroplast genomes, which was highly consistent with that estimated using DNA fragments (Li *et al.* 2016b; Ding *et al.* 2020). Moreover, the origin time of subg. *Cyathophora* (6.79 Mya) in the EL3 estimated by Xie *et al.* (2020a) was also approximately consistent with that (5.19 Mya) inferred using a constant substitution rate (Li *et al.* 2019). We thus set the root node of the EL3 at 16.97 Mya (spanning from 12.95 at the 2.5 % quantile to 19.85 at the 97.5 % quantile with the SD of 1.915) (Xie *et al.* 2020a); and set the crown node of subg. *Cyathophora* at 5.19 Mya (spanning from 3.56 at the 2.5 % quantile to 6.82 at the 97.5 % quantile with the S0044 of 0.83). Divergence time estimations were performed under an uncorrelated lognormal relaxed clock by using the complex GTR+I+G substitution model selected by jModelTest 2.1.10 (Darriba *et al.* 2012). Tree prior was specified as a speciation Yule process. Bayesian searches for tree topologies and node ages were run for two times each from a random starting tree, sampling every 1000th of 10 million MCMC generations. The first 20 % of cycles were discarded as burn-in and the post-burn-in trees were used to compute the divergence time. Effective sample size (ESS) values were well larger than 200 to confirm consensus of chains to a stationary distribution in Tracer v1.6 (Bouckaert *et al.* 2014). The maximum clade credibility (MCC) tree with common ancestor was produced using TreeAnnotator v1.8.4 (Bouckaert *et al.* 2014). Finally, MCC tree with ages for each node and their 95 % credible intervals was displayed in FigTree (http://tree.bio.ed.ac.uk/software/figtree).

### Ancestral area reconstruction

Ten geographic areas were delimited for *Allium* species: A: North America, B: Europe s.l., C: Africa, D: Western Irano-Turanian region including the Caucasian region, E: Arctic Asia, F: QTP s.l. (QTP, Tian Shan, Hengduan Mountains and the Himalayas), G: Northeast-Asian steppes (surrounded by Area AA in the North, QTP in the West; the border follows the Han River and further the Yangtze River in the South), H: Japan and Sakhalin, I: Subtropical Asia and J: Tropical Asia (Fig. 3A; **see****Supporting Information—Fig. S8**) (Hauenschild *et al.* 2017). Compared to the cpDNA data set, more species and subgenera of the EL3 were involved in the nrITS data set. We thus used the nrITS data set to estimate the ancestral geographic distributions of sect. *Daghestanica* with Reconstruct Ancestral States in Phylogenies (RASP) v3.0 under Bayesian Binary Method (BBM) (Yu *et al.* 2015). Geographical analyses were implemented using the nrITS consensus tree based on the F81 + G rate model. Each BBM analysis was set for 4 million generations using nine hot Markov chains and one cold chain with temperature increments of 0.1.

### Ecological niche difference tests

The 19 bioclimatic variables and altitude **[see****Supporting Information—Table S4****]** at 30 arc-second for the total 251 georeferenced coordinates **[see****Supporting Information—Table S5****]** were extracted from the WorldClim data (Hijmans *et al.* 2005), and those (Bio1 Annual mean temperature, Bio2 Mean diurnal range, Bio4 Temperature seasonality, Bio12 Annual precipitation, Bio15 Precipitation seasonality and altitude) exhibiting pairwise Pearson correlation coefficients *r* < 0.7 were finally used to conduct principle component analysis (PCA) using R (R Core Team 2018) princomp function. To further confirm the ecological differences among species, the density profiles were carried out using each bioclimatic variable in sm.density.compare function of R *sm* package (Bowman and Azzalini 2018), with 10 000 bootstrap to test significant difference.

## Results

### Summary of morphological traits of the Asian sect. *Daghestanica* species

The investigated morphological characters were summarized into **Table 1** using binary traits. The macromorphological traits sometimes have different states within species, while the micromorphological traits mostly were stable within species. The distinguishable traits used in this study including bulb shape; flower colour; perianth midvein; perianth shape (recurved 0, or not 1); nectary shape (concave 0, or not 1); projection shape (hood-like projection split 1, or not 0); stomatal apparatus (sunken/smooth 0, or not 1); outer arch of stomatal apparatus (raised 0, flat 1); shape of waxy ornaments in stomatal apparatus (absent 0, scale-like 1, block-like 2); cuticular papillates in leaf epidermis (absent 0 or not 1); seed shape and colour (long-ovate and black in 0); periclinal walls: shape and sculpturing; anticlinal walls: shape and sculpturing; dominant shape of testa cells; pollen shape; exine sculpture of pollen grains; leaf-blade shape in transection (linear 0, semiterete 1, terete 2, subterete 3, semiterete to narrow linear 4); palisade mesophyll (present 0, absent 1); spongy mesophyll (present 0, absent 1); distribution and orientation of vascular bundles (rings 0, other 1); shape of mesophyll cells (irregular 0, circular–ellipsoid 1); secondary wall thickening in cells adjacent to the phloem (present 0, absent 1); secondary wall thickening in cells adjacent to the xylem (present 0, absent 1) (**Table 1**). The details of these traits were described as follows.

**Table 1.** Summary of the morphological traits of Asian sect. *Daghestanica* species. *Allium rude*, RuD; *A. chrysanthum*, ChR; *A. chrysocephalum*, ChO; *A. herderianum*, HeR; *A. xichuanense*, XiC; *A. maowenense*, MaO; *A. xinlongense*, XiN. Missing data are coded as uncertain (–) in the character matrix.

Morphological traits	RuD	ChR	ChO	HeR	XiC	MaO	XiN
Bulb shape (cylindric 0; ovoid–cylindric 1; ovoid–globose to ovoid, 2)	0/1	0	0	2	2	1	0
Flower colour (pale yellow to greenish yellow 0, pale yellow to yellow 1, bright yellow 2, pale to bright yellow 3, white 4)	0	1	2	3	0	4	4
Perianth midvein (pale greenish 0, or reddish 1, or absent 2)	0	0	2	2	0	0/1	0/1
Perianth shape (recurved 0, or not 1)	1	1	0	0	1	1	1
Nectary shape (concave 0, or not 1)	0	1	0	0	0	0	0
Projection shape (hood-like projection split 1, or not 0)	0	0	0	0	0	0	1
Stomatal apparatus (sunken 0, or not 1)	0	0	0	0	0	0	0
Stomatal apparatus (smooth 0, or not 1)	1	0	1	1	1	0	0
Outer arch of stomatal apparatus (raised 0, flat 1)	0	1	0	0	0	1	0
Shape of waxy ornaments in stomatal apparatus (absent in 0, scale-like 1, block-like 2)	1	0	2	1	1	0	0
Cuticular papillates in leaf epidermis (absent 0, or not 1)	1	0	0	1	1	0	1
Seed shape and colour	0	0	0	0	0	0	0
Periclinal walls: shape and sculpturing	0	0	0	0	2	–	1
Anticlinal walls: shape and sculpturing	0	0	0	0	0	–	0
Dominant shape of testa cells	0	0	0	0	0	–	0
Pollen shape	0	0	0	1	0	0	–
Exine sculpture of pollen grains	0	0	1	1	0	0	–
Leaf-blade shape in transection (linear 0; semiterete 1; terete 2; subterete 3; semiterete to narrow linear 4)	0	2	0	4	1	0	3
Palisade mesophyll (present 0, absent 1)	1	1	1	1	1	1	1
Spongy mesophyll (present 0, absent 1)	0	0	0	0	0	0	0
Distribution and orientation of vascular bundles (rings 0, other 1)	0	0	0	0	0	1	0
Shape of mesophyll cells (irregular 0, circular–ellipsoid 1)	0	1	0	0	1	0	0
Secondary wall thickening in cells adjacent to the phloem (present 0, absent 1)	0	1	1	1	0	1	1
Secondary wall thickening in cells adjacent to the xylem (present 0, absent 1)	1	1	1	1	0	1	1

### Macromorphological traits in bulbs and flowers

The bulb shapes among Asian *Daghestanica* species show high diversity. Of which, *A. rude* has cylindric, sometimes narrowly ovoid–cylindric bulb; while cylindric to narrowly ovoid–cylindric in *A. chrysanthum*; cylindric, sometimes thickened at base in *A. chrysocephalum*; ovoid–globose to ovoid in *A. herderianum* and *A. xichuanense*; ovoid to narrowly so in *A. maowenense* and cylindric in *A. xinlongense***[see****Supporting Information—Fig. S1****]**. Perianth of *A. rude* (Fig. 1A and B) and *A. xichuanense* (Fig. 1F and G) is pale yellow to greenish yellow, with pale greenish midvein; whereas yellow to pale yellow perianth in *A. chrysanthum* (Fig. 1C); bright yellow perianth in *A. chrysocephalum* (Fig. 1D); pale to bright yellow perianth in *A. herderianum* (Fig. 1E); white perianth, with pale greenish or pale red midvein in *A. maowenense* (Fig. 1H); and white perianth with greenish or pale pinkish midvein in *A. xinlongense* (Fig. 1I). At the base of ovary among these species, only *A. chrysanthum* has no concave nectary (Fig. 1K), and the others show concave nectary covered by hood-like projections, which is highly specialized in *A. xinlongense* (Fig. 1J–P).

### Microsculpturing of leaves, seeds and pollen grains using SEM

The vocabularies we used to describe the microsculpturing of leaf epidermis, seed surface and pollen grain are drawn from the previous studies (Wilkinson 1980; Kosenko and Kudryashova 1995; Celep *et al.* 2012). The scanning electron micrographs for leaves, seeds and pollen grains are presented in **Supporting Information—Figs S2****–****S4**. Most microscopic traits in leaves, seeds and pollen grains are stable within species and just tiny difference was observed in *A. rude*. Sunken stomatal apparatus was found in all species of Asian sect. *Daghestanica*. Of these species, a significantly raised outer arch of stomatal apparatus was observed, except greatly flat ones in *A. chrysanthum* and *A. maowenense***[see****Supporting Information—Fig. S2****]**. Cuticular papillates are common in leaf epidermis for most species, except in *A. chrysanthum*, *A. chrysocephalum* and *A. maowenense***[see****Supporting Information—Fig. S2****]**. The scale-like waxy ornaments in stomatal apparatus were found in *A. rude*, *A. herderianum* and *A. xichuanense*, while block-like waxy ornaments in *A. chrysocephalum***[see****Supporting Information—Fig. S2****]**. However, no waxy ornaments were observed in the stomatal apparatus of *A. chrysanthum*, *A. maowenense* and *A. xinlongense***[see****Supporting Information—Fig. S2****]**. Noticeably, greatly smooth leaf epidermis and stomatal apparatus were detected in *A. chrysanthum* and *A. maowenense***[see****Supporting Information—Fig. S2****]**. The shape and colour of these species’ seeds are greatly similar, presenting narrowly long-ovate and black. The dominant shape of testa cells for these species mostly is tetra- to hexagonal. Periclinal walls are convex and ornamented by clear verrucate and granulose in all species. The sculpturing shapes are similar in most species, except the different ones in *A. xichuanense* and *A. xinlongense*. The smooth and highly same anticlinal walls are found in all species **[see****Supporting Information—Fig. S3****]**. The pollen shape mostly is long- elliptical, while somewhat of semicircle in *A. herderianum***[see****Supporting Information—Fig. S4****]**. The exine sculptures of pollen grains are larger circular or elliptic perforations in *A. chrysocephalum* and *A. herderianum*, while greatly smaller perforations and more striations in the other species **[see****Supporting Information—Fig. S4****]**.

### Paraffin anatomy of leaves

**Figure 2.**
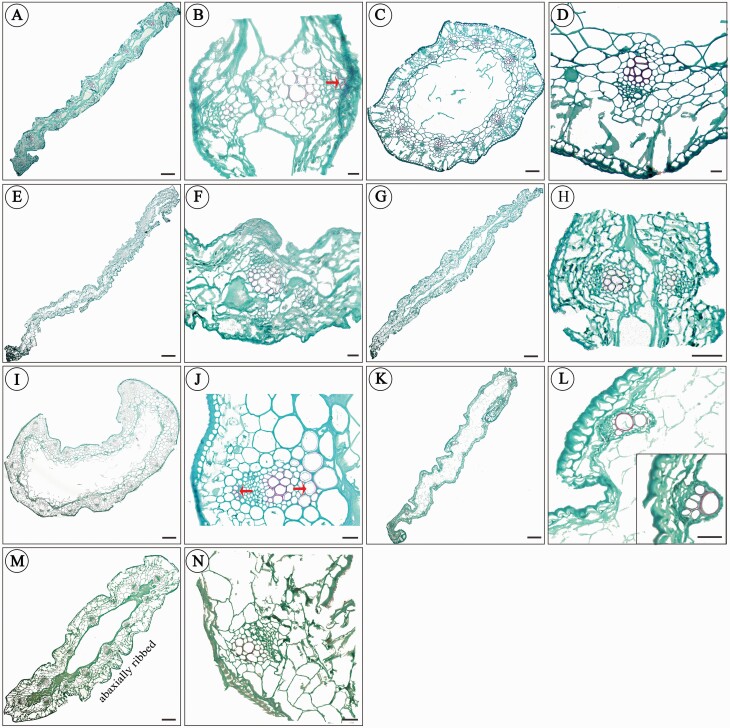
Paraffin transection of leaves for Asian sect. *Daghestanica* species. (A–B) *A. rude*; (C–D) *A. chrysanthum*; (E–F) *A. chrysocephalum*; (G–H) *A. herderianum*; (I–J) *A. xichuanense*; (K–L) *A. maowenense*; (M–N) *A. xinlongense*. The red arrows indicate the secondary wall thickening in cells adjacent to the phloem or xylem. The black arrows indicate the edges of linear leaves. Scale bar 1 mm.

The terminology describing leaf transverse section characters mainly followed Mashayekhi and Columbus (2014). The paraffin transection of leaves showed that *A. rude* (Fig. 2A), *A. chrysocephalum* (Fig. 2E) and *A. maowenense* (Fig. 2K) have linear leaf-blade shape; terete and fistulose leaves for *A. chrysanthum* (Fig. 2C); semiterete to semiterete-angled leaves for *A. xichuanense* (Fig. 2I); and subterete, fistulose, abaxially ribbed leaves for *A. xinlongense* (Fig. 2M). Palisade mesophyll is absent and spongy mesophyll is present in all species (Fig. 2). Each vascular bundle is surrounded by a layer of cells that forms a bundle sheath. Vascular bundles in most species are arranged in ring(s) and normally oriented, except in *A. maowenese* without xylem cells (Fig. 2l). Secondary wall thickening in cells adjacent to the phloem was found in *A. rude* and *A. xichuanense.* These cell walls are unevenly thickened and are mostly present in the adaxial half of the leaf in *A. rude* (Fig. 2B), while in the whole leaf in *A. xichuanense* (Fig. 2J). Multiple evenly thickened cell walls were also observed in cells near to the xylem in the whole leaf of *A. xichuanense* (Fig. 2J). Shape of mesophyll cells mostly is irregular in most species, while circular–ellipsoid in the leaves of *A. chrysanthum* and *A. xichuanense.* (Fig. 2).

### Phylogenetic relationships and networks

**Figure 3.**
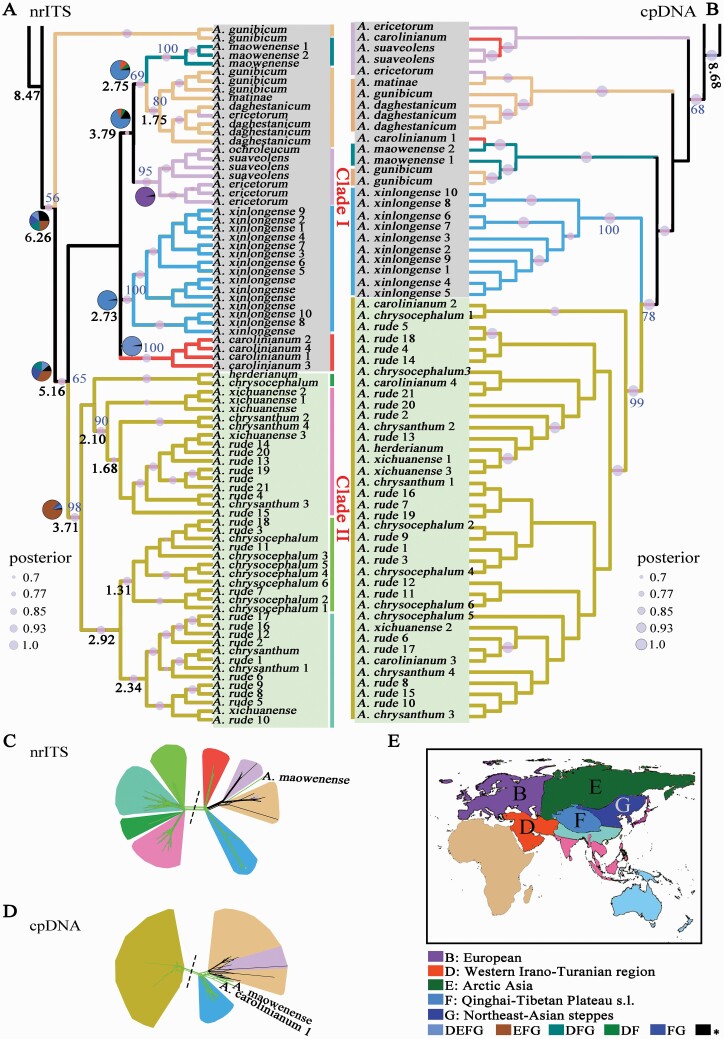
Evolutionary history reconstruction for sect. *Daghestanica* under the phylogenetic structure of the third evolutionary line (EL3) of *Allium***[see Supporting Information—****Figs S5****–****S8****]**. (A–B) Phylogenies, divergence time and biogeographic history of sect. *Daghestanica* based on the cpDNA fragments and nrITS sequences. The light blue circles indicate the posterior probability (PP > 0.7), and the blue numbers on the light blue circles represent the bootstrap values. The black numbers at the nodes indicate the mean divergence time (Mya; **see****Supporting Information—Figs S6****and****S7**). Two clades (I and II) of sect. *Daghestanica* are indicated by grey and light green. (C–D) Neighbour-net split graphs of sect. *Daghestanica* based on the nrITS and cpDNA sequences. The dotted lines indicate the split of two clades of sect. *Daghestanica*, and the colour of each subcluster corresponds to the species or species complex in the phylogenetic trees in A and B indicated by the same colour. The black and fluorescence-green solid lines in the networks, respectively, represent Caucasian–European and Asian species. (E) The legend of the biogeography used in B, and just relevant regions are shown (Hauenschild *et al.* 2017; **see****Supporting Information—Fig. S8**).

In this study, both nrITS and cpDNA (trnL–trnF and rpl32–trnL6) were used to reconstruct the phylogeny of the EL3. For all samples, the length of whole nrITS ranged from 397 to 639 bp with an alignment length of 506 bp; trnL–trnF varied the length from 163 to 572 bp with an alignment length of 185 bp because only part sequences were sequenced for Caucasian and European sect. *Daghestanica* species; and rpl32–trnL varied the length from 653 to 821 bp with an alignment length of 1077 bp. The molecular phylogenetic trees were shown in Fig. 3.

For the nrITS tree, subgenera *Butomissa* and *Cyathophora* formed the first two basal lineages and were successively sister to the complex clade including subgenera *Rhizirideum*, *Allium*, *Cepa*, *Reticulatobulbosa* and *Polyprason*, in which the phylogenetic relationships among subgenera were not well resolved with the lower support values **[see****Supporting Information—Fig. S5****]**. Interestingly, *A. carolinianum* belonging to subg. *Polyprason* sect. *Falcatifolia* was split into two groups: the samples collected in Qinghai (*A. carolinianum* 1–4) were clustered together and fell into sect. *Daghestanica* lineage (Fig. 3A); while the plants sampled in Tajikistan (*A. carolinianum* AJ250290) and Tibet (*A. carolinianum* GQ181097) were grouped together and fell into sect. *Falcatifolia***[see****Supporting Information—Fig. S5****]**. For sect. *Daghestanica*, all members were clustered into a monophyletic lineage (PP = 0.92; BP = 56). However, the phylogenetic relationships among the three geographical groups were distinctly inconsistent with the geography-based classification. Our nrITS tree indicated that sect. *Daghestanica* species were classified into two clades: the Clade I comprised Caucasian–European species and Asian *A. maowenense*, *Qinghai A. carolinianum* 1–4 and *A. xinlongense*, with six subclades divided to represent these species; the Clade II included Asian yellowish tepal species. In the Clade I, the first subclade only contained the two samples of Caucasian *A. gunibicum* (PP = 0.99, BP = 100) and were positioned at the basal of sect. *Daghestanica.* Although the relationships among the remaining five subclades were not well supported, the monophyly of each subclade was highly supported (PP > 0.90, BP > 80) (Fig. 3A). Among these five subclades, Qinghai *A. carolinianum* 1–4 (PP = 0.99, BP = 100), Asian *A. xinlongense* (PP = 0.98, BP = 100), European species (*A. suaveolens*, *A. ochroleucum* and *A. ericetorum* with PP = 0.99, BP = 95) and Asian *A. maowenense* (PP = 1.00, BP = 100), respectively, formed four reciprocally monophyletic lineages; while for the fifth subclade (PP = 0.98, BP = 80), all Caucasian species *A. gunibicum*, *A. matinae* and *A. daghestanicum* were grouped together, but one sample of European *A. ericetorum* also fell into Caucasian *A. daghestanicum* lineage (PP = 0.98, BP < 50). Asian *A. maowenense* was sister to the fifth subclade (PP = 0.97, BP = 69), and both then were sister to European subclade (PP = 0.76 and BP < 50) (Fig. 3A). In the Clade II, a highly paraphyletic pattern among the remaining five Asian species was recovered (Fig. 3A).

For the cpDNA tree, the relationships among subgenera of the EL3 were greatly consistent with the nrITS: subgenera *Butomissa* and *Cyathophora* were successively positioned at the basal and sister to the other subgenera. The relationships among the other subgenera were not well recovered with lower support values **[see****Supporting Information—Fig. S6****]**. As the nrITS tree shown, sect. *Daghestanica* species were clustered together in the cpDNA tree (PP = 0.99, BP = 68) and also split into two clades: the Clade I comprised Caucasian, European species and Asian *A. maowenense*, Tajikistan *A. carolinianum*, Qinghai *A. carolinianum* 1 and Asian *A. xinlongense*; the Clade II majorly included Asian yellowish tepal species (PP = 0.99; BP = 99) (Fig. 3B). In the Clade I, four subclades were recognized with no clearly geographical classification and poorly recovered phylogenetic relationships between them. Among these subclades, Asian *A. xinlongense* formed a monophyletic lineage (PP = 0.99; BP = 100); European subclade species (*A. ericetorum* and *A. suaveolens*) were grouped together with Tajikistan *A. carolinianum*; Caucasian subclade species (*A. matinae*, *A. gunibicum* and *A. daghestanicum*) were clustered together with European *A. ericetorum*; and Asian *A. maowenense* were joined together with Qinghai *A. carolinianum* 1 and Caucasian *A. gunibicum* (Fig. 3B). Inconsistent with the nrITS tree (Fig. 3A), *A. xinlongense* was sister to the Clade II (PP = 1.0, BP = 78), which included *A. carolinianum* 2–4, *A. chrysocephalum*, *A. chrysanthum*, *A. herderianum*, *A. rude* and *A. xichuanense* in the cpDNA tree (Fig. 3B).

Given the paraphyletic relationships among sect. *Daghestanica* species, our neighbour-net split graphs were used to further illuminate their evolutionary relationships. The genetic relationships of sect. *Daghestanica* species based on the neighbour-net network analyses were highly in agreement with the tree-like topologies both in the nrITS and cpDNA data sets (Fig. 3). In the nrITS-based network, two same clades were detected as the nrITS tree shown (Fig. 3A and C). The Clade I included Caucasian and European species, and Asian *A. maowenense*, *A. xinlongense* and Qinghai *A. carolinianum* 1–4, in which European *A. ericetorum* and Asian *A. maowenense* were clustered together with Caucasian species; and only *A. gunibicum* and *A. ericetorum* presented a paraphyletic pattern. The Clade II mainly comprised Asian yellowish tepal species with no assured species relationships and were further split into four subclades, as the nrITS tree shown (Fig. 3A and C). For the cpDNA-based network, two similar clades were also observed. Different from the nrITS neighbour-net network, Qinghai *A. carolinianum* 2–4 fell into Asian group and presented a paraphyletic pattern, while Qinghai *A. carolinianum* 1 was clustered together with Asian *A. maowenense*. A closer relationship for Caucasian and European species was recovered, as well as a closer distance between *A. xinlongense* and Caucasus–Europe–*A. maowenense*–*A. carolinianum* 1, which was incongruent with the cpDNA tree topology but agreed with the nrUTS tree topology (Fig. 3). Noticeably, Asian species in the Clade II were linked by a greatly narrowly meshed networks compared to those in the nrITS (Fig. 3C and D).

### Divergence time estimation and historical biogeography

The BEAST-derived chronogram tracked the crown age of the EL3 back to the early Miocene: in the nrITS, around 14.84 Mya [95 % highest posterior density (HPD), 18.40–10.86 Mya]; in the cpDNA around 15.51 Mya (95 % HPD, 19.15–11.59 Mya) **[see****Supporting Information—Figs S6****and****S7****]**. These resulting root time estimations of the EL3 were highly consistent with the used calibrated time 16.97 Mya (95 % HPD, 19.85–12.95 Mya) (Xie *et al.* 2020a). The divergence time of subg. *Cyathophora* was dated back to ~5.57 Mya (95 % HPD, 6.99–3.94 Mya) in the nrITS tree, and around 6.17 Mya (95 % HPD, 6.55–3.51 Mya) in the cpDNA tree **[see****Supporting Information—Figs S6****and****S7****]**; which was highly consistent with our calibration time 5.19 Mya (95 % HPD, 6.82–3.56 Mya), as well as the time (6.79 Mya) estimated by Xie *et al.* (2020a). All these resulting time estimations indicated a suitable calibration for the divergence time of the EL3. We found that the intra-subgenus divergence in the EL3 mostly began around 8 Mya both in the nrITS and cpDNA trees **[see****Supporting Information—Figs S6****and****S7****]**. The divergence time estimations based on the cpDNA sequences had a much border 95 % HPD than those in the nrITS tree **[see****Supporting Information—Figs S6****and****S7****]**. Given the more confused phylogenetic signals in the cpDNA tree compared with the nrITS tree (e.g. *A. carolinianum*, *A. maowenense*) (Fig. 3A and B), we used the nrITS result to assess the divergence of sect. *Daghestanica*. The age of the crown node of sect. *Daghestanica* was tracked back to the Late Miocene, around 6.26 Mya (95 % HPD, 8.76–3.83 Mya). The divergence between the two clades of sect. *Daghestanica* was dated back to the Early Pliocene, around 5.16 Mya (95 % HPD, 5.20–3.17 Mya) (Fig. 3B). The interspecific and intraspecific divergence with sect. *Daghestanica* mainly occurred around 4–3 Mya **[see****Supporting Information—Fig. S7****]**. In the Clade I, the divergence between European and Caucasian subclades including Asian *A. maowenense* and European *A. ericetorum* begun around 3.79 Mya (95 % HPD, 5.82–1.88 Mya); and the divergence between Asian *A. maowenense* and Caucasian subclade including European *A. ericetorum* occurred around 2.75 Mya (95 % HPD, 4.48–1.18 Mya). The divergence of the Clade II comprising Asian yellowish tepal species *A. chrysocephalum*, *A. chrysanthum*, *A. herderianum*, *A. rude* and *A. xichuanense* mainly occurred around 3.71 Mya (95 % HPD in the nrITS, 5.35–2.13 Mya).

The RASP-based historical biogeography using the nrITS data set clearly showed that the ancient geographical distributions of the root of the EL3 located in the QTP and the adjacent region (with PP = 1.0 at the root node: 64.77 % FG + 25.04 % F + 5.12 % G + 5.07 % unknown; with PP = 1.0 at the second crown node of the EL3: 62.41 % FG + 15.53 % EFG + 11.13 % F + 10.93 % unknown) **[see****Supporting Information—Fig. S8****]**. The result inferred that the ancient population of sect. *Daghestanica* probably was located in the QTP and the adjacent region or however unknown region, with the first two crown nodes mostly involving in the QTP and the adjacent region, or relatively larger proportion of unknown region (PP = 0.92 at the first crown node: 24.50 % FG + 18.60 % DFG + 17.19 % EFG + 13.05 % DEFG + 26.66 % unknown; PP = 0.93 at the second crown node: 33.08 % FG + 25.21 % EFG + 17.34 % DFG + 13.21 % DEFG + 11.16 % unknown) (Fig. 3A and E). The ancient population of Asian yellowish tepal species was also inferred to distribute in the QTP and the adjacent region (PP = 0.99 at the node 4: 86.02 % FG + 5.73 % EFG + 5.59 % F + 2.66 % unknown) (Fig. 3C).

### Ecological differences among Asian species

**Figure 4.**
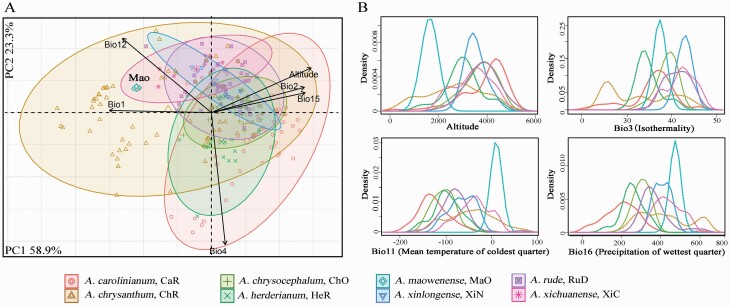
Ecological niche differentiation among the Asian sect. *Daghestanica* species. (A) Scatter plots of the first two components representing environmental niche differentiation among the investigated taxa in the PCA. (B) Four representatives of density profiles for the investigated taxa (other density profiles can be seen in **Supporting Information—Fig. S9**).

Given the paraphyletic relationships among Asian species, we further investigated their ecological niche differentiation. Over 10 georeferenced localities were sampled for each species, except for *A. maowenense* and *A. xinlongense* with fewer ones due to their narrow distribution and/or the fewer confirmed distribution localities **[see****Supporting Information—Table S5****]**. However, when *A. maowenense* and *A. xinlongense* were removed in the species-combined data set, there were little effects on the results of PCA and density profiles. Thus, all species were included to perform ecological difference tests. The PCA result of six bioclimatic variables uncovered two components (PC1 and PC2) that cumulatively explained 82.2 % of variation (Fig. 4A). The scatter plot presented that these species can be divided into three subgroups along the PC1 and PC2. The first group included *A. chrysanthum*, *A. xichuanense* and *A. maowenense*, determined by Bio1, Bio2, Bio12, Bio15 and altitude. The second group comprised *A. carolianum*, *A. chrysocephalum* and *A. herderianum*, defined by Bio2, Bio4, Bio15 and altitude. The third group contained *A. rude* and *A. xinlongense*, discriminated by Bio2, Bio12, Bio15 and altitude. Towards this result, the first and second groups can be reciprocally differentiated by Bio12 and Bio4, while the third group mostly occupies the intermediate ecological niches between those of the first and second groups, and Bio12 and altitude are the major determinants (Fig. 4A). The density profiles of each bioclimatic variable presented clearly realized niche difference both in the most occupied densities and parameter spans (all *P* < 0.001) among these species **[see****Supporting Information—Fig. S9****]**. Of these density and span comparisons, altitude presented an increased order in which *A. maowenense* < *A. chrysanthum* < *A. herderianum* ≤ *A. carolinianum* < *A. xinlongense* < *A. xichuanense* < *A. rude* ≤ *A. chrysocephalum* < *A. carolinianum* (Fig. 4B). Of the temperature-correlated variables (Bio1–Bio11; **see****Supporting Information—Fig. S9**), the similar density profiles in Bio1, Bio6, Bio9, Bio10 and Bio11 showed an increased order in which *A. carolinianum* < *A. chrysocephalum* ≤ *A. herderianum* < *A. rude* < *A. xinlongense* < *A. xichuanense* ≤ *A. chrysanthum* < *A. maowenense* (Fig. 4B). For the precipitation-related variables (Bio12–Bio19; **see****Supporting Information—Fig. S9**), the similar density patterns also presented an increased order in which *A. carolinianum* < *A. herderianum* < *A. chrysocephalum* ≤ *A. carolinianum < A. rude* < *A. xinlongense* ≤ *A. xichuanense* < *A. maowenense* < *A. carolinianum* (Fig. 4B).

## Discussion

In this study, we reconstructed the phylogeny of the third evolutionary line (EL3) of the genus *Allium* based on the nrITS and cpDNA (concatenation of trnL–trnF and rpl32–trnL), to further elaborate the evolutionary history among the three disjunctive geographical groups of sect. *Daghestanica*. Our study provides an insight to understand the origin and diversification of sect. *Daghestanica* across Eurasia.

### Insights for the evolutionary history of the EL3

Our molecular phylogeny of the EL3 was highly consistent with previous studies (Friesen *et al.* 2006; Li *et al.* 2010). Subgenera *Butomissa* and *Cyathophora* are successively positioned at the first two basal clades of and the remaining subgenera form an admixed clade **[see****Supporting Information—Figs S5****and****S6****]**. The divergence time estimations indicated that the EL3 originated around 15 Mya both in the cpDNA (15.51 Mya with 95 % HPD, 11.59–19.15 Mya) **[see****Supporting Information—Fig. S6****]** and nrITS (14.81 Mya with 95 % HPD, 10.86–18.40 Mya) **[see****Supporting Information—Fig. S7****]**, and the divergence between subgenera occurred around 13–8 Mya **[see****Supporting Information—Figs S6****and****S7****]**. These divergence time estimations approximately fell into the time range revealed by Xie *et al*. (2020), indicating the second calibration was applicable in this study. The RASP-based biogeography inference strongly indicated that the EL3 most likely originated in the QTP and the adjacent region (PP = 1.0) **[see****Supporting Information—Fig. S8****]**. The divergence of intra-subgenus within the EL3 mostly began around 8–7 Mya and 4–3 Mya **[see****Supporting Information—Fig. S7****]**, coinciding with the dramatic uplift of the QTP around these periods (Harrison *et al.* 1992; Shi *et al.* 1998; Liu and Dong 2013; Li *et al.* 2014). We thus inferred that the QTP movement could have contributed much to the EL3 evolution (Wen *et al.* 2014; Li *et al.* 2016a; Xie *et al.* 2019a; Ding *et al.* 2020).

The field observations found *A. carolinianum* collected in Qinghai (*A. carolinianum* 1–4) and Tibet (GenBank number GQ181097) have the highly similar morphology **[see****Supporting Information—Fig. S10****]**, while they were clustered into different sections: Qinghai *A. carolinianum* 1–4 were clustered together with sect. *Daghestanica* species, and Tibet and Tajikistan *A. carolinianum* were clustered together and sister with sect. *Falcatifolia* species **[see****Supporting Information—Fig. S5****]**. Such paraphyletic pattern seems prevalent in subgenera of the EL3 (Friesen *et al.* 2006; Li *et al.* 2010; Hauenschild *et al.* 2017), for example in sect*. Sikkimensa* (Xie *et al.* 2019a) and in the *A. saxatile* group (Seregin *et al.* 2015). The prevalence of genetic and morphological paraphyly in the EL3 admonishes us that the classifications of subgenera and sections in the EL3 should be studied with great care, and the interspecific relationships in young lineages of the EL3 should be reconstructed at population level using multiple genes. In this study, more unresolved relationships were found in the cpDNA tree than those in the nrITS tree **[see****Supporting Information—Figs S5****and****S6****]**. We thought that the limited genetic information provided by the two concatenated cpDNA fragments could have caused the unstable topology of the cpDNA tree. Sun *et al.* (2020) have provided the chromosome-level genome assembly of *A. sativum*, which would bring new opportunity for the phylogeny of *Allium* by using transcriptomes and whole-genome resequencing.

### Out of the QTP and evolutionary radiation across Eurasia for sect. *Daghestanica*

According to the geographic distribution pattern, sect. *Daghestanica* species can be classified into three disjunctive distribution groups: European, Caucasian and Asian. However, no such classification was recovered in our molecular phylogenetic analyses because Asian *A. maowenense* and European *A. ericetorum* fell into Caucasian lineage both in the cpDNA and nrITS trees (Fig. 3). The inter-clade and interspecific relationships were also not well resolved by the tree-based phylogeny (Fig. 3A and B). However, network methods provide a valuable tool for such uncertain phylogenetic science (Hudson and Bryant 2006). Our uncorrected_*P*-distance-based networks indicated that Asian *A. maowenense*, *A. xinlongense* and *A. caroliniarum* have a closer genetic relationship with Caucasian–European group; and Asian *A. maowenense* and Caucasian species could be at the base of sect. *Daghestanica* with the longer branch length and closer *P*-distance between them (Fig. 3C and D), which is highly congruent with their morphological similarity in densely many flowers per inflorescence and whitish tepals, especially the reddish midvein both in Asian *A. maowenense* and Caucasian *A. daghestanicum*.

The RASP-based biogeography inference strongly indicated (PP > 0.9) that the ancient population of sect. *Daghestanica* was located in the QTP and the adjacent region or unknown region (Fig. 3A and E), because of the unresolved evolutionary relationships among the three geographically disjunctive groups (Fig. 3). Ding *et al.* (2020) has indicated that the alpine flora of the eastern QTP has continuously existed far longer than any other alpine flora on Earth. Considering the morphology polymorphisms in Caucasian group (i.e. the morphology similarity between Caucasian *A. daghestanicum* and Asian *A. maowenense*, as well as between Caucasian *A. matinae* and European group) and the beginning anthesis in autumn to adapt the Mediterranean climate, the possibility with regard to the origin and diversification of the three disjunctive geographical groups of sect. *Daghestanica* can be inferred with the most parsimonious way: Caucasus and Europe distributions of this section are secondary; the migration of Asian ancestor into Caucasian and resulted in further diversification either from Caucasus to Europe, or from Caucasus to Europe then back to Caucasus (Fig. 3A and E). In such situation, just Caucasian group needs to adapt the Mediterranean climate by transferring the beginning anthesis in summer to in autumn, rather than Asian and European groups both transferred the beginning anthesis in autumn to in summer. The divergence of sect. *Daghestanica* occurred around the Late Miocene (~6.26 Mya), which followed the QTP uplift around 8–7 Mya. We inferred the QTP uplift could have acted as significant geographical barrier between the ancient region (Asia) and the secondary region (Caucasus–Europe).

Our molecular phylogenies (Fig. 3) and macromorphological traits (Fig. 1; **see****Supporting Information—Fig. S11**) strongly indicated two group of Asian sect. *Daghestanica*: the whitish tepal group (*A. maowenense*, *A. xinlongense* and *A. carolinianum* 1–4) and the yellowish tepal group (*A. chrysocephalum*, *A. chrysanthum*, *A. herderianum*, *A. rude* and *A. xichuanense*). Our results showed that the yellowish tepal group originated in the QTP and the adjacent region around 3.71 Mya (Fig. 3A), which coincides with the QTP uplift around 4–3 Mya. Our ecological comparisons among Asian species revealed a greatly different ecological niches for these species (Fig. 4). We thus speculated that the heterogeneous geomorphological and environmental mosaic in the QTP and the adjacent region due to the QTP uplift could have promoted the rapid radiation of this group. Such evolutionary radiation in the QTP is common (e.g. Wen *et al.* 2014; Sun *et al.* 2017), especially in *Allium* evolution (e.g. Huang *et al.* 2014; Li *et al.* 2016a; Li *et al.* 2016b; Hauenschild *et al.* 2017; Xie *et al.* 2019a). Though the yellowish tepal species can be clearly delimited using morphological traits and ecological niches (Table 1; Fig. 4), a paraphyletic pattern was recovered in the molecular analyses (Fig. 3). Aside from the limited variation information of the selected DNA sequences causing this paraphyletic pattern, incomplete lineage sorting and introgressive hybridization should also be taken into account (Fu *et al.* 2021).

### Morphology evolution for Asian sect. *Daghestanica* species

Although the whitish tepal species and yellowish tepal species in Asian sect. *Daghestanica* belong to different evolutionary clades (Fig. 3), the relatively conservative microsculpturing in leaves, seeds and pollen grains are still preserved among all these species, probably suggesting the evolutionary homology due to the shared ancestor. For example, the highly similar leaf-transection structure (Fig. 2), tetra- to hexagonal testa cells, shapes of periclinal and anticlinal walls **[see****Supporting Information—Fig. S3****]** and pollen grain traits **[see****Supporting Information—Fig. S4****]**. Among these morphological traits, the leaf polymorphisms are the most attention-getting. The paraffin transection of leaves showed that the linear leaves occur in both fistular-leaved or solid-leaved species, such as in *A. rude*, *A.* chrysocephalum, *A. herderianum* and *A. maowenense* (Fig. 2); while the terete (*A. chrysanthum*), semiterete (*A. xichuanense*) and subterete (*A. xinlongense*) leaves are presented only in fistular-leaved species (Fig. 2). Such pattern seemingly suggests that the leaf shape might be not related with the formation of fistular or solid leaves. It has been documented that the process of fistular leaf formation involves programmed cell death (Wang 2010; Zhu *et al.* 2017). The vascular bundles in Asian sect. *Daghestanica* mostly are arranged in ring(s) and normally oriented, except for *A. maowenense* without a ring structure (Fig. 2). Our field investigations found that the leaves of *A. maowenense* mostly are flexuous **[see****Supporting Information—Fig. S11F****]**, but the other congeners mostly have erect-angled leaves **[see****Supporting Information—Fig. S2****]**. The density profiles indicated that *A. maowenense* has a mild habitat characterized by the low-altitude and high-temperature/precipitation compared to the rather rigorous alpine habitats for the other congeners (Fig. 4B). We thus inferred that the structure of vascular bundles might have a supporting role for the leaves, and the evolution of fistular leaves might be coupled with environmental factors. For example, secondary wall thickening in cells just was observed in *A. rude* and *A. xichuanense* (Fig. 2), which could also be related with stress tolerance in the alpine habitats (Guo *et al.* 2017).

### Identification key for eight Asian sect. *Daghestanica* species

1 Perianth yellow ..................... 2

-   Perianth white ..................... 3

2 Leaves linear ..................... 4

-   Leaves not linear ..................... *5*

4 Perianth bright yellow; apex recurved .......... *A. chrysocephalum*

-   Perianth pale yellow to greenish yellow; apex not recurved ..................... *A. rude*

5 Leaves terete, fistulose ..................... A. chrysanthum

-   Leaves semiterete ..................... 6

6 Leaves semiterete to narrow linear; perianths apex recurved ..................... *A. herderianum*

-   Leaves semiterete to semiterete-angled, fistulose ..................... *A. xichuanense*

3 Leaves linear ..................... *7*

-   Leaves subterete, abaxially ribbed ..................... A. xinlongense

7 Leaves broadly linear, usually falcate, in Qinghai Province ..................... *A. carolinianum*

- Leaves linear, flexuous ..................... *A. maowense*

## Conclusions

Using nrITS and two combined cpDNA genes, we found two clades for sect. *Daghestanica*, which is incongruent with the classification based on the three disjunctive geographical distributions. One clade includes Europe–Caucasus–Asia species, and the other clade contains Asian yellowish tepal species. The divergence time estimations and biogeography inference suggested that Asian ancestor migrated into Caucasus and Europe around the Late Miocene, and further caused divergence and speciation in these secondary areas. Moreover, our morphological and ecological investigations indicated an evolutionary radiation for Asian yellowish tepal species in the QTP and the adjunctive region around 4–3 Mya, mostly due to the heterogeneous geomorphological and environmental mosaic in the QTP.

## Supporting Information

The following additional information is available in the online version of this article—

**Figure S1.** Bulb characters of the Asian sect. *Daghestanica*. (A–E) *A. rude*; (F) *A. chrysanthum*; (G) *A. chrysocephalum*; (H) *A. herderianum*; (I) *A. xichuanense*; (J) *A. maowenense*; (K) *A. xinlongense*.

**Figure S2.** Scanning electron micrographs of leaves of the Asian sect. *Daghestanica*. (A–D) *A. rude*; (E–F) *A. chrysanthum*; (G–H) *A. chrysocephalum*; (I–J) *A. herderianum*; (K–L) *A. xichuanense*; (M–N) *A. maowenense*; (O–P) *A. xinlongense*.

**Figure S3.** Seed characters of the Asian sect*. Daghestanica*. (A, G–I) *A. rude*; (B, J–K) *A. chrysanthum*; (C, L) *A. chrysocephalum*; (D, M–N) *A. herderianum*; (E, O) *A. xichuanense*; (F, P) *A. xinlongense*.

**Figure S4.** Pollen characters of the Asian sect. *Daghestanica*. (A–B) *A. rude*; (C–D) *A. chrysanthum*; (E–F) *A. chrysocephalum*; (G–H) *A. herderianum*; (I–J) *A. xichuanense*; (K–L) *A. maowenense*. Scale bar 10 μm.

**Figure S5.** Phylogeny of the third evolutionary line (EL3) of the genus *Allium* based on the nrITS sequences. The Bayesian inference (BI) tree was shown due to the similar topology between the BI and the maximum parsimony (MP) tree. The species sequences downloaded from the GenBank are marked by the accession number. The posterior probability (PP) is indicated by the light blue circle with PP > 0.7, and the bootstrap values of the maximum likelihood (ML) are indicated by the number on the blue circles.

**Figure S6.** Phylogeny of the third evolutionary line (EL3) of the genus *Allium* based on the concatenation of trnL–trnF and rpl32–trnL. The Bayesian inference (BI) tree was shown due to the similar topology between the BI and the maximum parsimony (MP) tree. The posterior probability (PP > 0.7) is shown by the light blue circle; the numbers on the PP circle are the bootstrap values (BP) of the maximum likelihood (ML) tree. The black numbers at the nodes indicate the mean time and 95 % highest posterior density (HPD), which was calibrated by the time of the root of the EL3 and the crown node of the subg. *Cyathophora*. The species sequences downloaded from the GenBank are listed in **Supporting Information—Table S5**.

**Figure S7.** Divergence time estimation of the evolutionary line 3 (EL3) of the genus *Allium* based on the nrITS sequences using the root node of the EL3 (16.097 million years ago) and the crown node of the subg. *Cytahophora* (5.19 million years ago) to calibrate the time. The tree topology is same with that in **Supporting Information—Fig. S6**.

**Figure S8.** RASP-based geographical distribution of the third evolutionary line (EL3) of the genus *Allium* based on the nrITS sequences.

**Figure S9.** Density plots of the Asian sect. *Daghestanica* species and *A. carolinianum* for 20 bioclimatic variables. Significance values of differences among species for each variable, tested using 10 000 bootstraps. In most density profiles, *A. carolinianum* have two peaks representing samples in the Qinghai and Tibet and XinJiang Province.

**Figure S10.** Morphological comparisons among the populations of *A. carolinianum* collected in different locations. (A–B) Plants collected in Tibet, with the nrITS sequence number GQ181097. (C–E) Plants collected in Qinghai Province, coded with *A. carolinianum* 1–4.

**Figure S11.** Leaf comparisons among the seven Asian sect. *Daghestanica*. (A) *A. rude*; (B) *A. chrysanthum*; (C) *A. chrysocephalum*; (D) *A. herderianum*; (E) *A. xichuanense*; (F) *A. maowenense*; (G–H) *A. xinlongense*.

**Table S1.** The detailed information for the samples used in this study.

**Table S2.** GenBank number newly produced in the study.

**Table S3.** Bioclimatic variables extracted from WorldClim data set.

**Table S4.** The detailed information for 251 vetted localities of *A. carolianum* (CaR), *A. rude* (RuD), *A. chrysanthum* (ChR), *A. xichuanense* (XiC), *A. herderianum* (HeR), *A. chrysocephalum* (ChO), *A. maowenense* (MaO) and *A. xinlongense* (XiN).

**Table S5.** GenBank number of the cpDNA downloaded in this study.

plab017_suppl_Supplementary_Materials

## Data Availability

The newly generated sequences were submitted in NCBI (https://www.ncbi.nlm.nih.gov/) with the GenBank accession numbers shown in **Supporting Information—Table S2**; and the GenBank accession numbers of downloaded sequences from NCBI are shown in **Supporting Information—Fig. S6** and **Supporting Information—Table S5**.

## Notes

**Evolution & Diversity. Chief Editor:** Jeremy Beaulieu
