# Supplementary material for: Out of the Qinghai–Tibetan Plateau and rapid radiation across Eurasia for Allium section Daghestanica (Amaryllidaceae)
Source: AoB Plants. 2021 Apr 14;13(3):plab017. doi: 10.1093/aobpla/plab017 (PMC8152445; doi:10.1093/aobpla/plab017)
Supplement: plab017_suppl_Supplementary_Materials [file plab017_suppl_supplementary_materials.pdf]

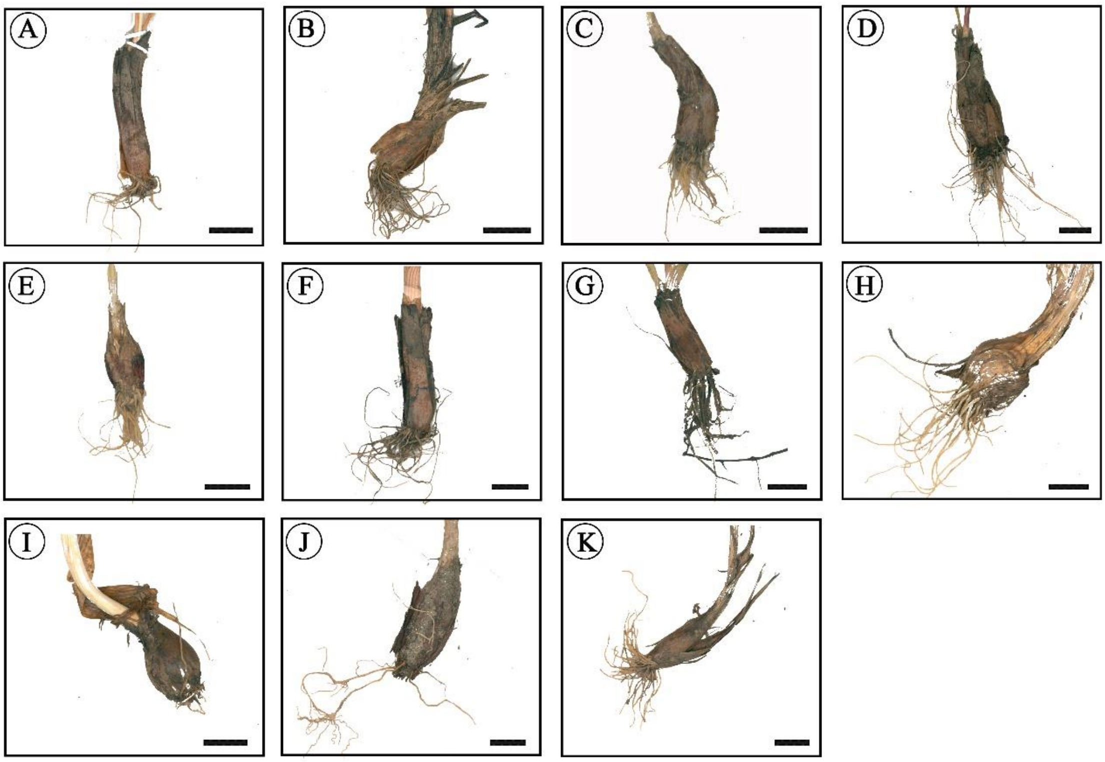

Fig. S1 Bulb characters of the Asian sect. *Daghestanica*. (A–E) *A. rude*; (F) *A. chrysanthum*; (G) *A. chrysocephalum*; (H) *A. herderianum*; (I) *A. xichuanense*; (J) *A. maowenense*; (K) *A. xinlongense*.

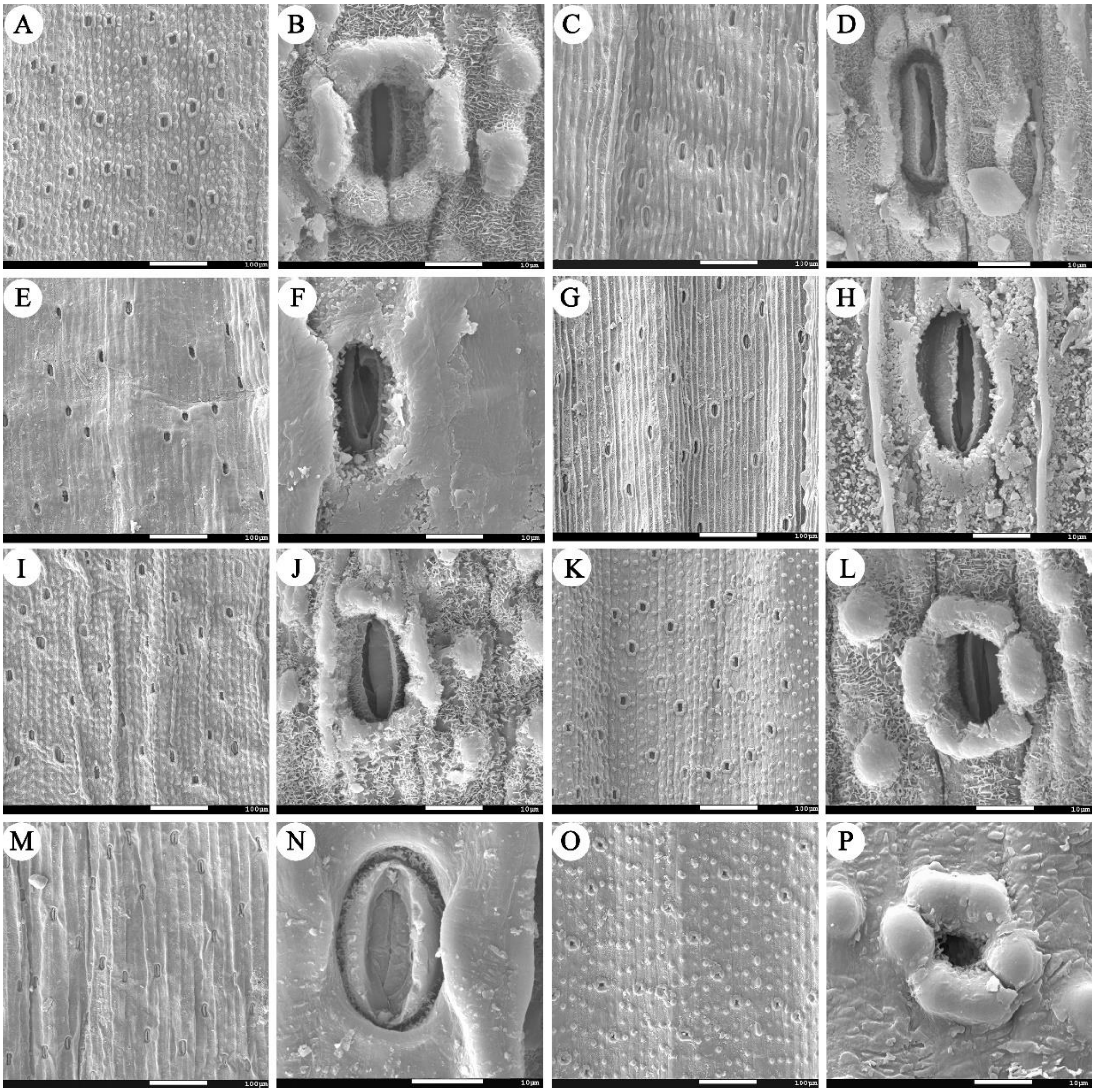

Fig. S2 Scanning electron micrographs of leaves of the Asian sect. *Daghestanica*. (A–D) *A. rude*; (E–F) *A. chrysanthum*; (G–H) *A. chrysocephalum*; (I–J) *A. herderianum*; (K–L) *A. xichuanense*; (M–N) *A. maowenense*; (O–P) *A. xinlongense*.

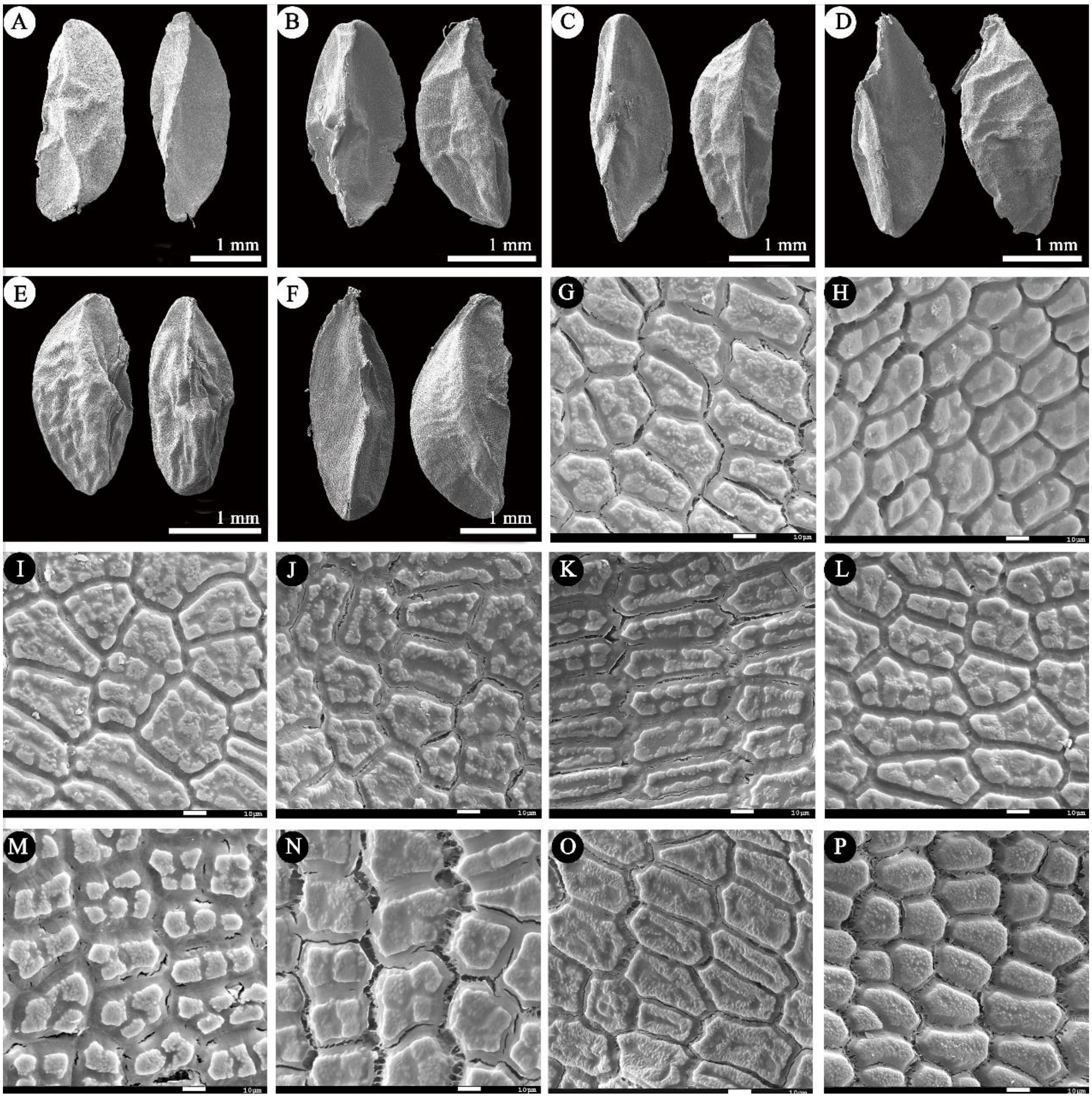

Fig. S3 Seed characters of the Asian sect. *Daghestanica*. (A, G–I) *A. rude*; (B, J–K) *A. chrysanthum*; (C, L) *A. chrysocephalum*; (D, M–N) *A. herderianum*; (E, O) *A. xichuanense*; (F, P) *A. xinlongense*.

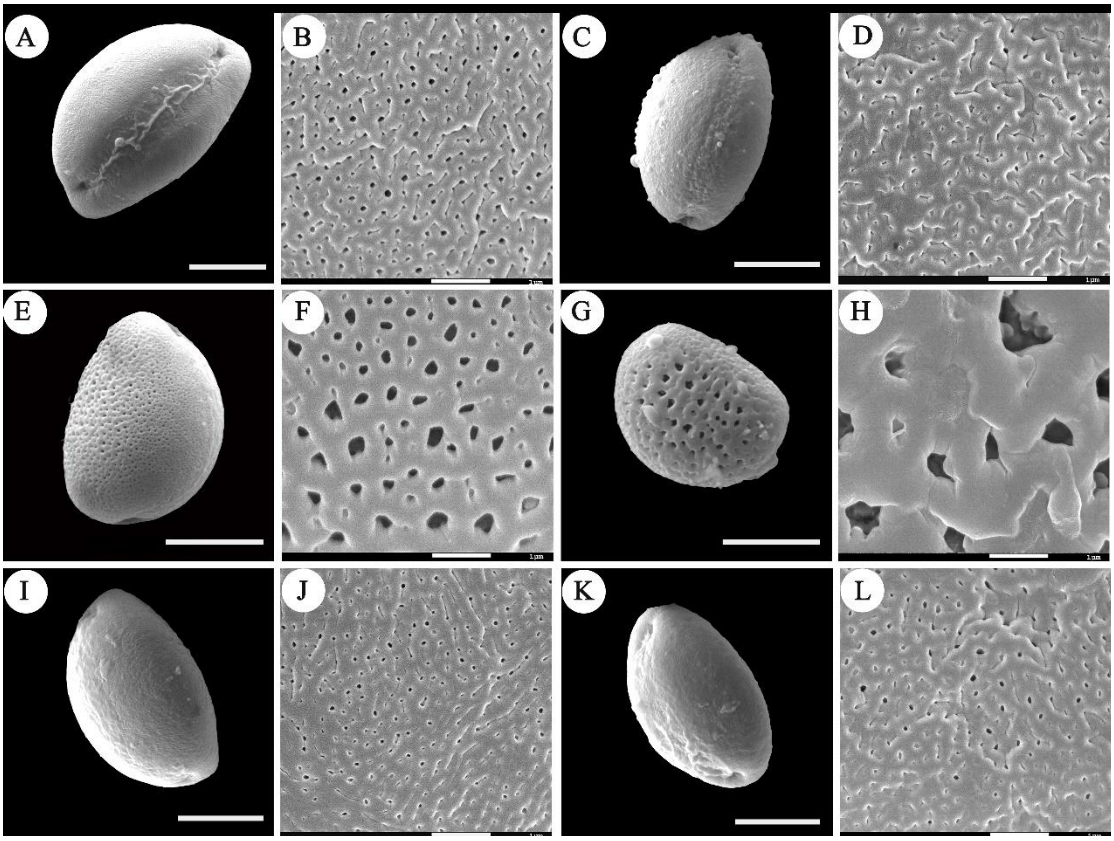

Fig. S4 Pollen characters of the Asian sect. *Daghestanica*. (A–B) *A. rude*; (C–D) *A. chrysanthum*; (E–F) *A. chrysocephalum*; (G–H) *A. herderianum*; (I–J) *A. xichuanense*; (K–L) *A. maowenense*. Scale bar 10 µm.

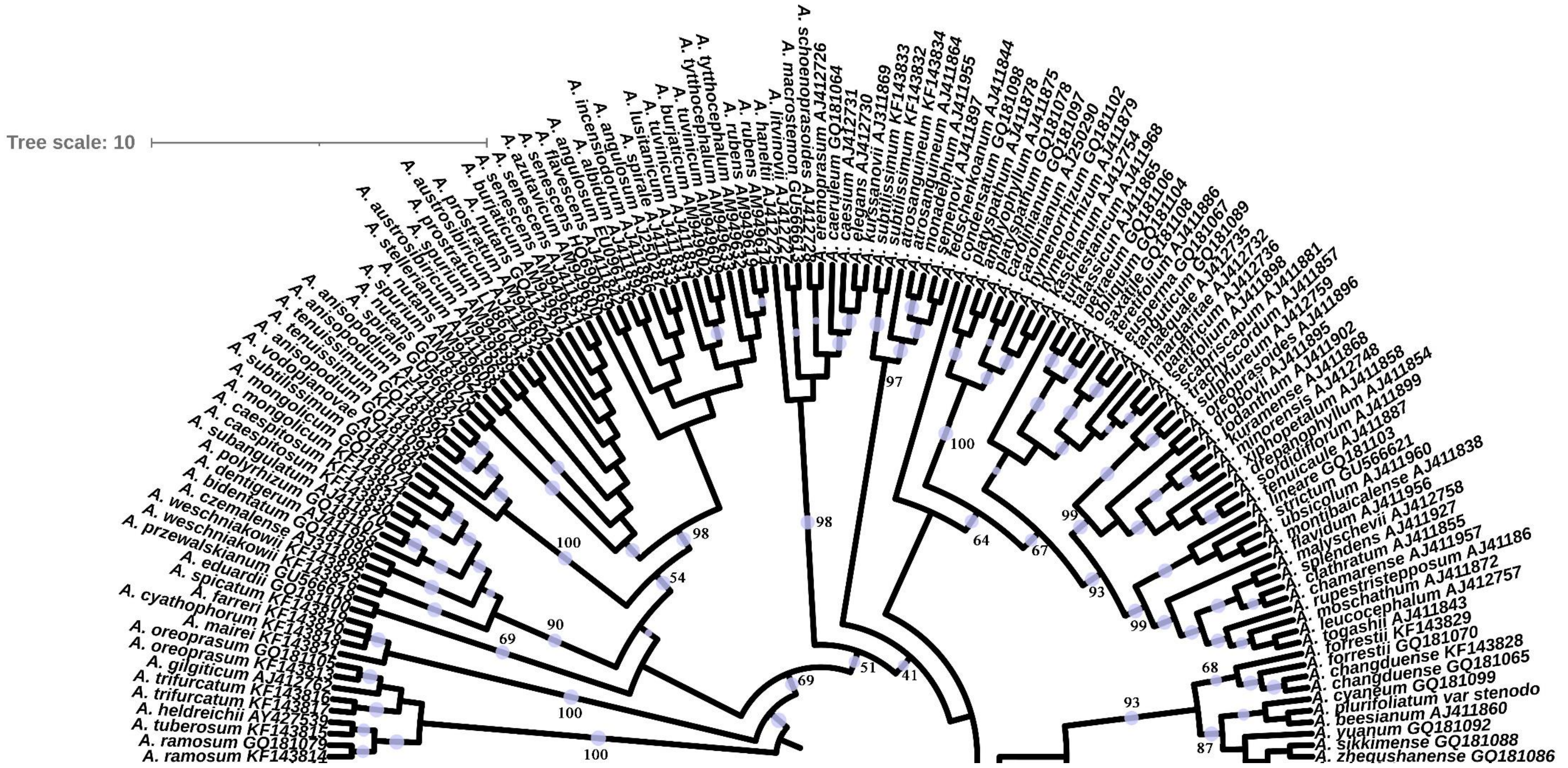
Tree scale: 10
A. zhegushanense GQ181086
A. sikkimense GQ181088
A. yuanum GQ181092
A. beesianum AJ411860
A. plurifoliatum var stenodo
A. cyaneum GQ181099
A. changduense GQ181065
A. changduense KF143828
A. forrestii GQ181070
A. forrestii KF143829
A. togashii AJ411843
A. leucocephalum AJ412757
A. moschathum AJ411872
A. chamarense AJ411957
A. clathratum AJ411855
A. splendens AJ411927
A. flavidum AJ411956
A. montibaicalense AJ411838
A. ubsicolum AJ411960
A. strictum GU566621
A. lineare GQ181103
A. tenuicaule AJ411887
A. ramosum KF143814
A. ramosum GQ181079
A. tuberosum KF143815
A. heldreichii AY427539
A. trifurcatum KF143817
A. trifurcatum KF143816
A. gilgiticum AJ412762
A. oreoprasum KF143813
A. oreoprasum GQ181105
A. mairei KF143870
A. cyathophorum KF143819
A. farreri KF143810
A. spicatum GQ181100
A. eduardii GU566676
100
98
97
100
98
64
67
99
93
99
54
90
69
100
100
51
41
69
68
93
87

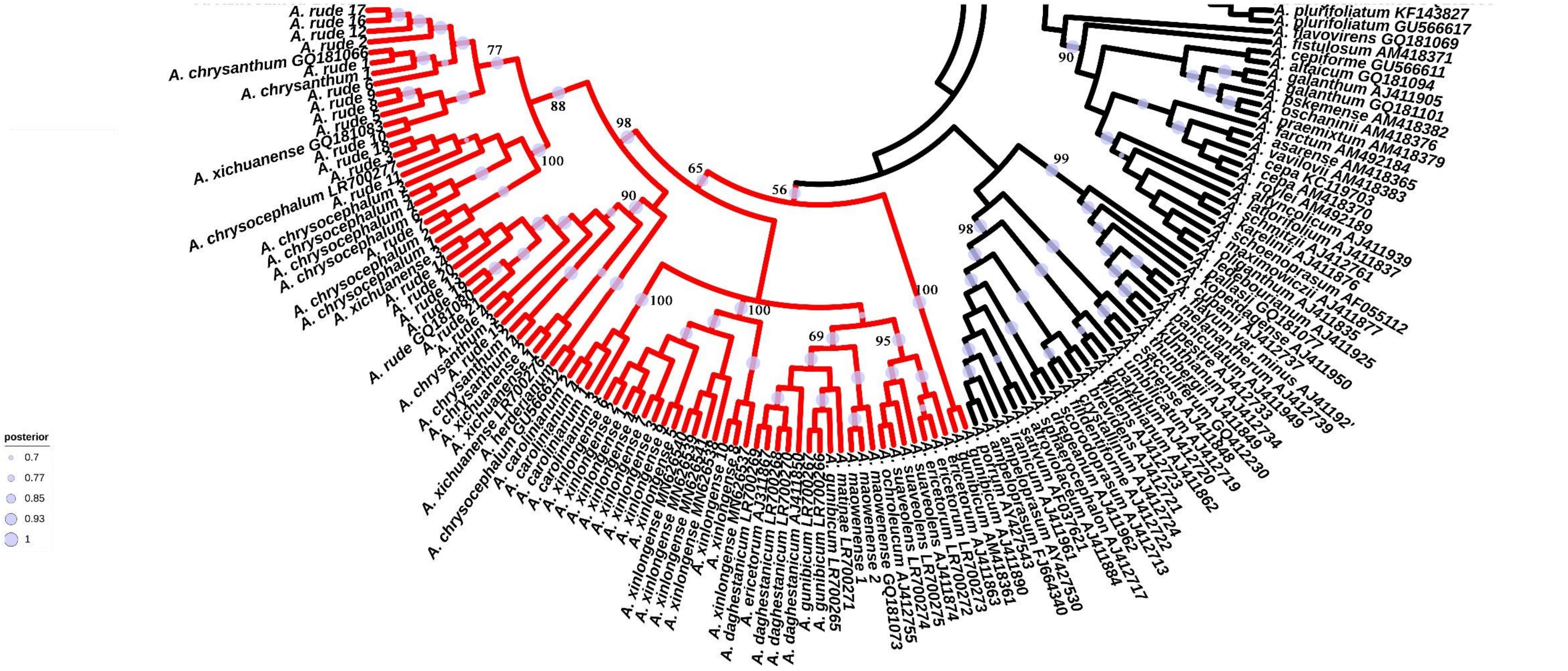

Fig. S5 Phylogeny of the third evolutionary line (EL3) of the genus *Allium* based on the nrITS sequences. The Bayesian inference (BI) tree was shown due to the similar topology between the BI and the maximum parsimony (MP) tree. The species sequences downloaded from the GenBank are marked by the accession number. The posterior probability (PP) are indicated by the light blue circle with PP > 0.7, and the bootstrap values of the ML are indicated by the number on the blue circles.

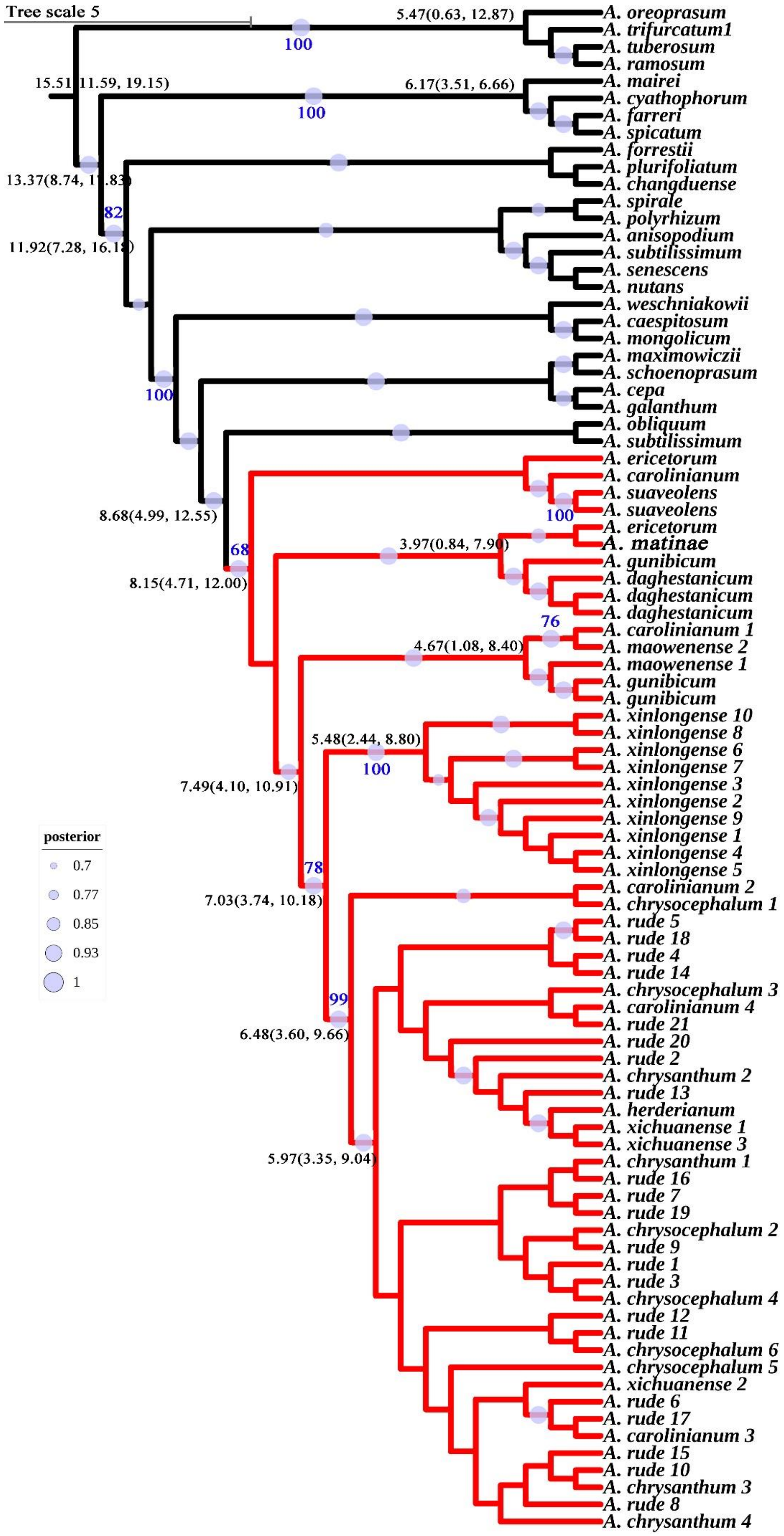
Tree scale 5
15.51(11.59, 19.15)
13.37(8.74, 11.83)
11.92(7.28, 16.1)
8.68(4.99, 12.55)
8.15(4.71, 12.00)
7.49(4.10, 10.91)
7.03(3.74, 10.18)
6.48(3.60, 9.66)
5.97(3.35, 9.04)
5.47(0.63, 12.87)
6.17(3.51, 6.66)
3.97(0.84, 7.90)
4.67(1.08, 8.40)
5.48(2.44, 8.80)
100
100
82
100
100
68
76
100
78
99
posterior
0.7
0.77
0.85
0.93
1
A. oreoprasum
A. trifurcatum1
A. tuberosum
A. ramosum
A. mairei
A. cyathophorum
A. farreri
A. spicatum
A. forrestii
A. plurifoliatum
A. changduense
A. spirale
A. polyrhizum
A. anisopodium
A. subtilissimum
A. senescens
A. nutans
A. weschniakowii
A. caespitosum
A. mongolicum
A. maximowiczii
A. schoenoprasum
A. cepa
A. galanthum
A. obliquum
A. subtilissimum
A. ericetorum
A. carolinianum
A. suaveolens
A. suaveolens
A. ericetorum
A. matinae
A. gunibicum
A. daghestanicum
A. daghestanicum
A. daghestanicum
A. carolinianum 1
A. maowenense 2
A. maowenense 1
A. gunibicum
A. gunibicum
A. xinlongense 10
A. xinlongense 8
A. xinlongense 6
A. xinlongense 7
A. xinlongense 3
A. xinlongense 2
A. xinlongense 9
A. xinlongense 1
A. xinlongense 4
A. xinlongense 5
A. carolinianum 2
A. chrysocephalum 1
A. rude 5
A. rude 18
A. rude 4
A. rude 14
A. chrysocephalum 3
A. carolinianum 4
A. rude 21
A. rude 20
A. rude 2
A. chrysanthum 2
A. rude 13
A. herderianum
A. xichuanense 1
A. xichuanense 3
A. chrysanthum 1
A. rude 16
A. rude 7
A. rude 19
A. chrysocephalum 2
A. rude 9
A. rude 1
A. rude 3
A. chrysocephalum 4
A. rude 12
A. rude 11
A. chrysocephalum 6
A. chrysocephalum 5
A. xichuanense 2
A. rude 6
A. rude 17
A. carolinianum 3
A. rude 15
A. rude 10
A. chrysanthum 3
A. rude 8
A. chrysanthum 4

Fig. S6 Phylogeny of the third evolutionary line (EL3) of the genus *Allium* based on the concatenation of trnL-trnF and rpl32-trnL. The Bayesian inference (BI) tree was shown due to the similar topology between the BI and the maximum parsimony (MP) tree. The posterior probability (PP > 0.7) is shown by the light blue circle; the numbers on the PP circle are the bootstrap values (BP) of the maximum likelihood (ML) tree. The black numbers at the nodes indicate the mean time and 95% highest posterior density (HPD), which was calibrated by the time of the root of the EL3 and the crown node of the subg. *Cyathophora*. The species sequences downloaded from the GenBank are listed in Table S5.

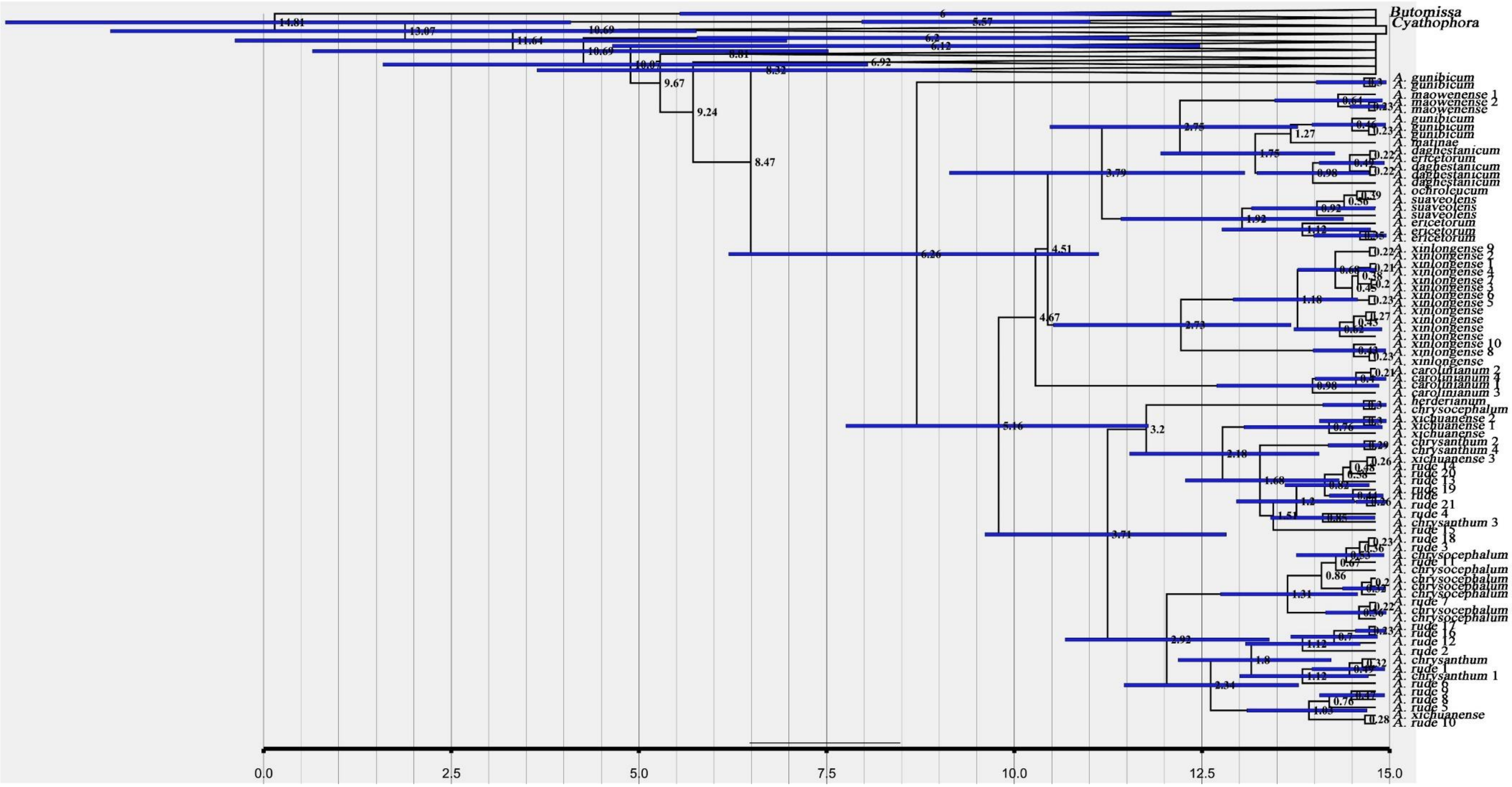
Butomissa
Cyathophora
A. gunibicum
A. gunibicum
A. maowenense 1
A. maowenense 2
A. maowenense
A. gunibicum
A. gunibicum
A. gunibicum
A. matinae
A. daghestanicum
A. ericetorum
A. daghestanicum
A. daghestanicum
A. daghestanicum
A. ochroleucum
A. suaveolens
A. suaveolens
A. suaveolens
A. ericetorum
A. ericetorum
A. ericetorum
A. xinlongense 9
A. xinlongense 2
A. xinlongense 1
A. xinlongense 4
A. xinlongense 7
A. xinlongense 3
A. xinlongense 6
A. xinlongense 5
A. xinlongense
A. xinlongense
A. xinlongense
A. xinlongense
A. xinlongense 10
A. xinlongense 8
A. xinlongense
A. carolinianum 2
A. carolinianum 4
A. carolinianum 1
A. carolinianum 3
A. herderianum
A. chrysocephalum
A. xichuanense 2
A. xichuanense 1
A. xichuanense
A. chrysanthum 2
A. chrysanthum 4
A. xichuanense 3
A. rude 14
A. rude 20
A. rude 13
A. rude 19
A. rude
A. rude 21
A. rude 4
A. chrysanthum 3
A. rude 15
A. rude 18
A. rude 3
A. chrysocephalum
A. rude 11
A. chrysocephalum 3
A. chrysocephalum 5
A. chrysocephalum 4
A. chrysocephalum 6
A. rude 7
A. chrysocephalum 2
A. chrysocephalum 1
A. rude 17
A. rude 16
A. rude 12
A. rude 2
A. chrysanthum
A. rude 1
A. chrysanthum 1
A. rude 6
A. rude 9
A. rude 8
A. rude 5
A. xichuanense
A. rude 10
0.0
2.5
5.0
7.5
10.0
12.5
15.0

Fig. S7 Divergence time estimation of the evolutionary line 3 (EL3) of the genus *Allium* based on the nrITS sequences using the root node of the EL3 (16.097 million years ago) and the crown node of the subg. *Cytahophora* (5.19 million years ago) to calibrate the time. The tree topology is same with that in Fig. S6.

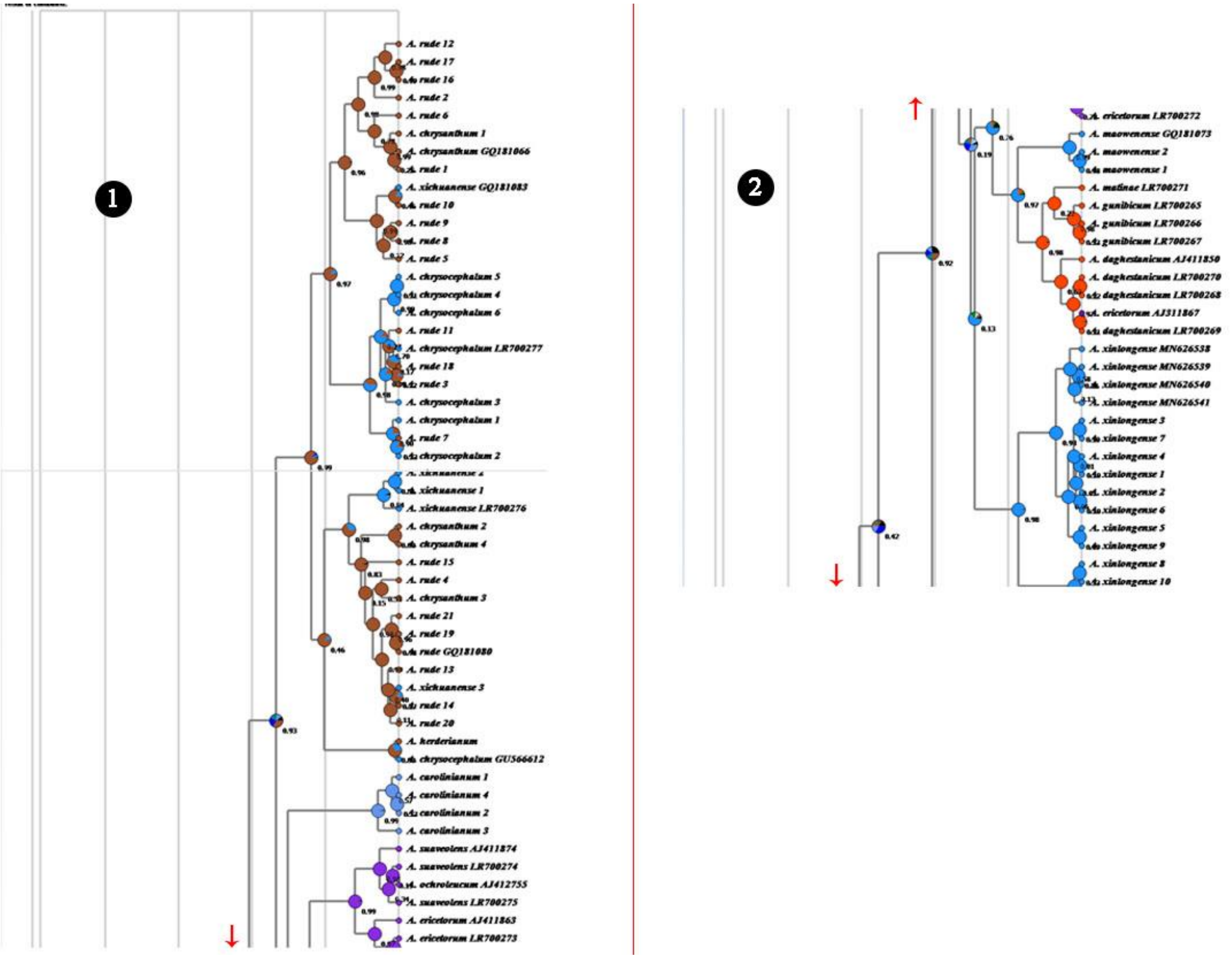

1
A. rude 12
A. rude 17
A. rude 16
A. rude 2
A. rude 6
A. chrysanthum 1
A. chrysanthum GQ181066
A. rude 1
A. xichuanense GQ181083
A. rude 10
A. rude 9
A. rude 8
A. rude 5
A. chrysocephalum 5
A. chrysocephalum 4
A. chrysocephalum 6
A. rude 11
A. chrysocephalum LR700277
A. rude 18
A. rude 3
A. chrysocephalum 3
A. chrysocephalum 1
A. rude 7
A. chrysocephalum 2
A. xichuanense 1
A. xichuanense LR700276
A. chrysanthum 2
A. chrysanthum 4
A. rude 15
A. rude 4
A. chrysanthum 3
A. rude 21
A. rude 19
A. rude GQ181080
A. rude 13
A. xichuanense 3
A. rude 14
A. rude 20
A. herderianum
A. chrysocephalum GU566612
A. carolinianum 1
A. carolinianum 4
A. carolinianum 2
A. carolinianum 3
A. suaveolens AJ411874
A. suaveolens LR700274
A. ochroleucum AJ412755
A. suaveolens LR700275
A. ericetorum AJ411863
A. ericetorum LR700273
2
A. ericetorum LR700272
A. maowenense GQ181073
A. maowenense 2
A. maowenense 1
A. malinae LR700271
A. gunibicum LR700265
A. gunibicum LR700266
A. gunibicum LR700267
A. daghestanicum AJ411850
A. daghestanicum LR700270
A. daghestanicum LR700268
A. ericetorum AJ311867
A. daghestanicum LR700269
A. xinlongense MN626538
A. xinlongense MN626539
A. xinlongense MN626540
A. xinlongense MN626541
A. xinlongense 3
A. xinlongense 7
A. xinlongense 4
A. xinlongense 1
A. xinlongense 2
A. xinlongense 6
A. xinlongense 5
A. xinlongense 9
A. xinlongense 8
A. xinlongense 10

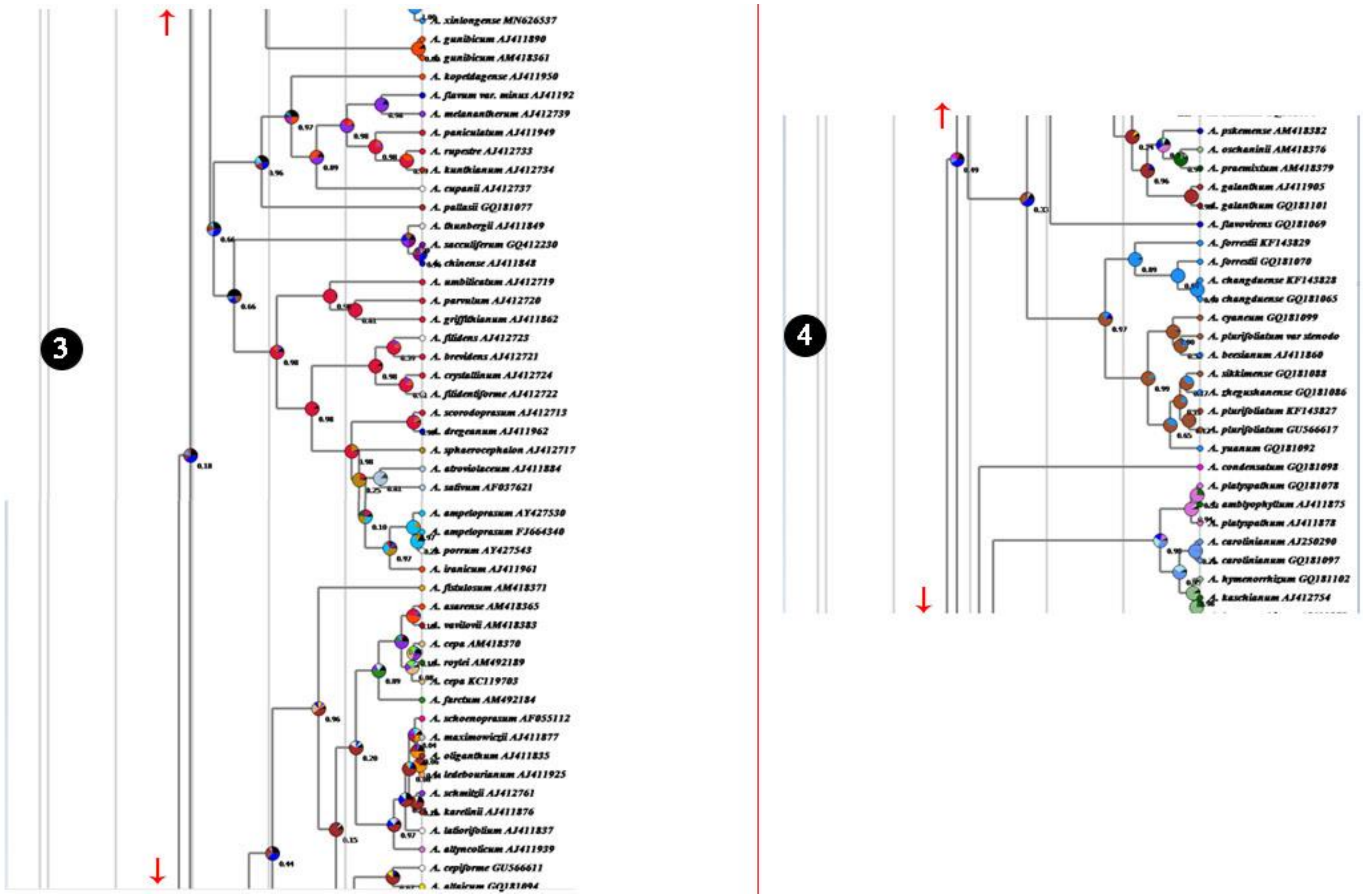
3
A. xinlongense MN626537
A. gunibicum AJ411890
A. gunibicum AM418361
A. kopetdagense AJ411950
A. flavum var. minus AJ41192
A. melananthenum AJ412739
A. paniculatum AJ411949
A. rupestre AJ412733
A. kunthianum AJ412734
A. cupanii AJ412737
A. pallasii GQ181077
A. thunbergii AJ411849
A. sacculiferum GQ412230
A. chinense AJ411848
A. umbilicatum AJ412719
A. parvulum AJ412720
A. griffithianum AJ411862
A. filidens AJ412723
A. brevidens AJ412721
A. crystallinum AJ412724
A. filidentiforme AJ412722
A. scorodoprasum AJ412713
A. dregeanum AJ411962
A. sphaerocephalon AJ412717
A. atroviolaceum AJ411884
A. sativum AF037621
A. ampeloprasum AY427530
A. ampeloprasum FJ664340
A. porrum AY427543
A. iranicum AJ411961
A. fistulosum AM418371
A. asarense AM418365
A. vavilovii AM418383
A. cepa AM418370
A. roylei AM492189
A. cepa KC119703
A. farctum AM492184
A. schoenoprasum AF055112
A. maximowiczii AJ411877
A. oliganthum AJ411835
A. ledebourianum AJ411925
A. schmitzii AJ412761
A. karelinii AJ411876
A. latiorifolium AJ411837
A. altyncolicum AJ411939
A. cepiforme GU566611
A. altaicum GQ181094
4
A. pskemense AM418382
A. oschaninii AM418376
A. praemixtum AM418379
A. galanthum AJ411905
A. galanthum GQ181101
A. flavovirens GQ181069
A. forrestii KF143829
A. forrestii GQ181070
A. changduense KF143828
A. changduense GQ181065
A. cyaneum GQ181099
A. plurifoliatum var stenodo
A. beesianum AJ411860
A. sikkimense GQ181088
A. zhegushanense GQ181086
A. plurifoliatum KF143827
A. plurifoliatum GU566617
A. yuanum GQ181092
A. condensatum GQ181098
A. platyspathum GQ181078
A. amblyophyllum AJ411875
A. platyspathum AJ411878
A. carolinianum AJ250290
A. carolinianum GQ181097
A. hymenorrhizum GQ181102
A. kaschianum AJ412754

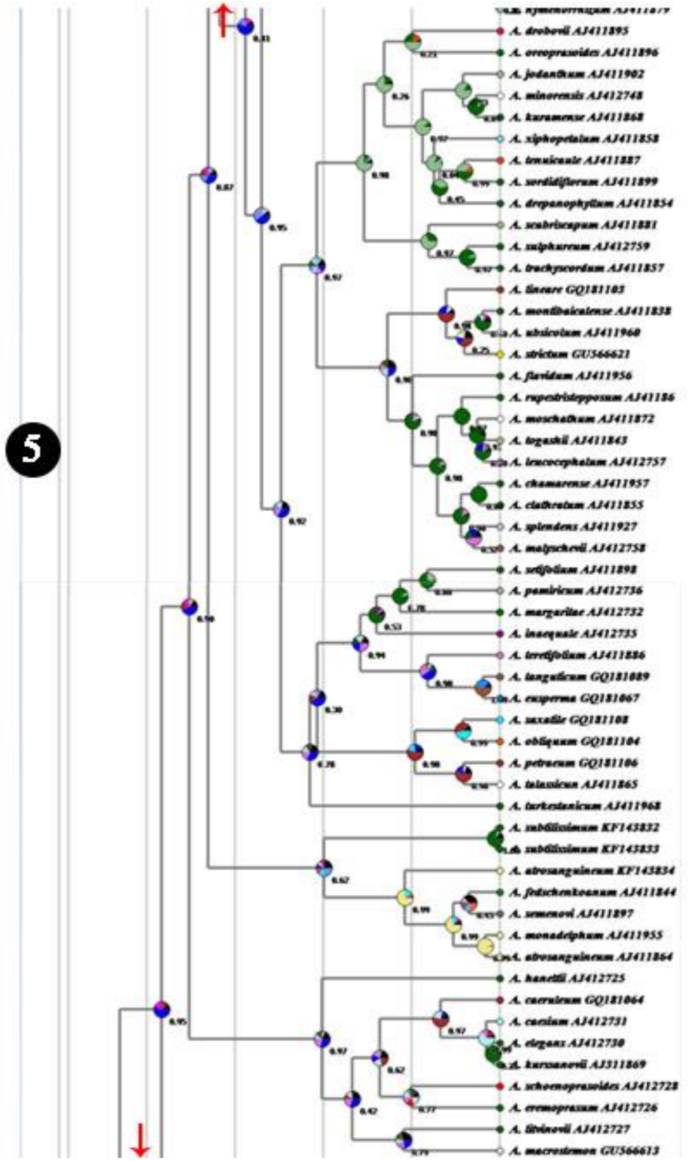
5
A. drobovii AJ411895
A. oreoprasoides AJ411896
A. jodanthum AJ411902
A. minorensis AJ412748
A. kuramense AJ411868
A. xiphopetalum AJ411858
A. tenuicaule AJ411887
A. sordidiflorum AJ411899
A. drepanophyllum AJ411854
A. scabriscapum AJ411881
A. sulphureum AJ412759
A. trachyscordum AJ411857
A. lineare GQ181105
A. montibaicalense AJ411838
A. ubsicolum AJ411960
A. strictum GU566621
A. flavidum AJ411956
A. rupestristepposum AJ41186
A. moschatum AJ411872
A. togashii AJ411843
A. leucocephalum AJ412757
A. chamarense AJ411957
A. clathratum AJ411855
A. splendens AJ411927
A. malyschevii AJ412758
A. setifolium AJ411898
A. pamiricum AJ412736
A. margaritae AJ412732
A. inaequale AJ412735
A. teretifolium AJ411886
A. tanguticum GQ181089
A. eusperma GQ181067
A. saxatile GQ181108
A. obliquum GQ181104
A. petraeum GQ181106
A. talassicum AJ411865
A. turkestanicum AJ411968
A. subtilissimum KF143832
A. subtilissimum KF143833
A. atrosanguineum KF143834
A. fedschenkoanum AJ411844
A. semenovi AJ411897
A. monadelphum AJ411955
A. atrosanguineum AJ411864
A. hanelti AJ412725
A. caeruleum GQ181064
A. caesium AJ412731
A. elegans AJ412730
A. kurssanovii AJ311869
A. schoenoprasoides AJ412728
A. eremoprasum AJ412726
A. litvinovii AJ412727
A. macrostemon GU566613

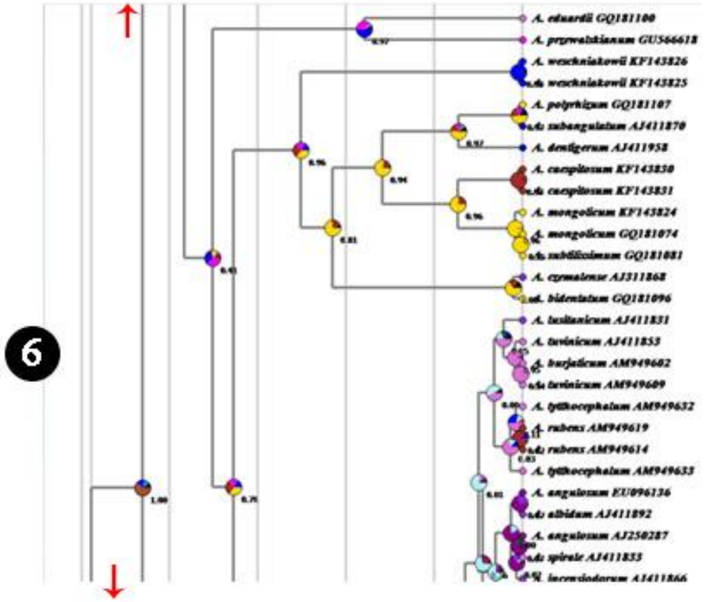
6
A. eduardii GQ181100
A. przewalskianum GU566618
A. weschniakowii KF143826
A. weschniakowii KF143825
A. polyrhizum GQ181107
A. subangulatum AJ411870
A. dentigerum AJ411958
A. caespitosum KF143830
A. caespitosum KF143831
A. mongolicum KF143824
A. mongolicum GQ181074
A. subtilissimum GQ181081
A. czymaiense AJ311868
A. bidentatum GQ181096
A. lusitanicum AJ411831
A. tuvinicum AJ411853
A. burjaticum AM949602
A. tuvinicum AM949609
A. tytthocephalum AM949632
A. rubens AM949619
A. rubens AM949614
A. tytthocephalum AM949633
A. angulosum EU096136
A. albidum AJ411892
A. angulosum AJ250287
A. spirale AJ411833
A. incensiodorum AJ411866

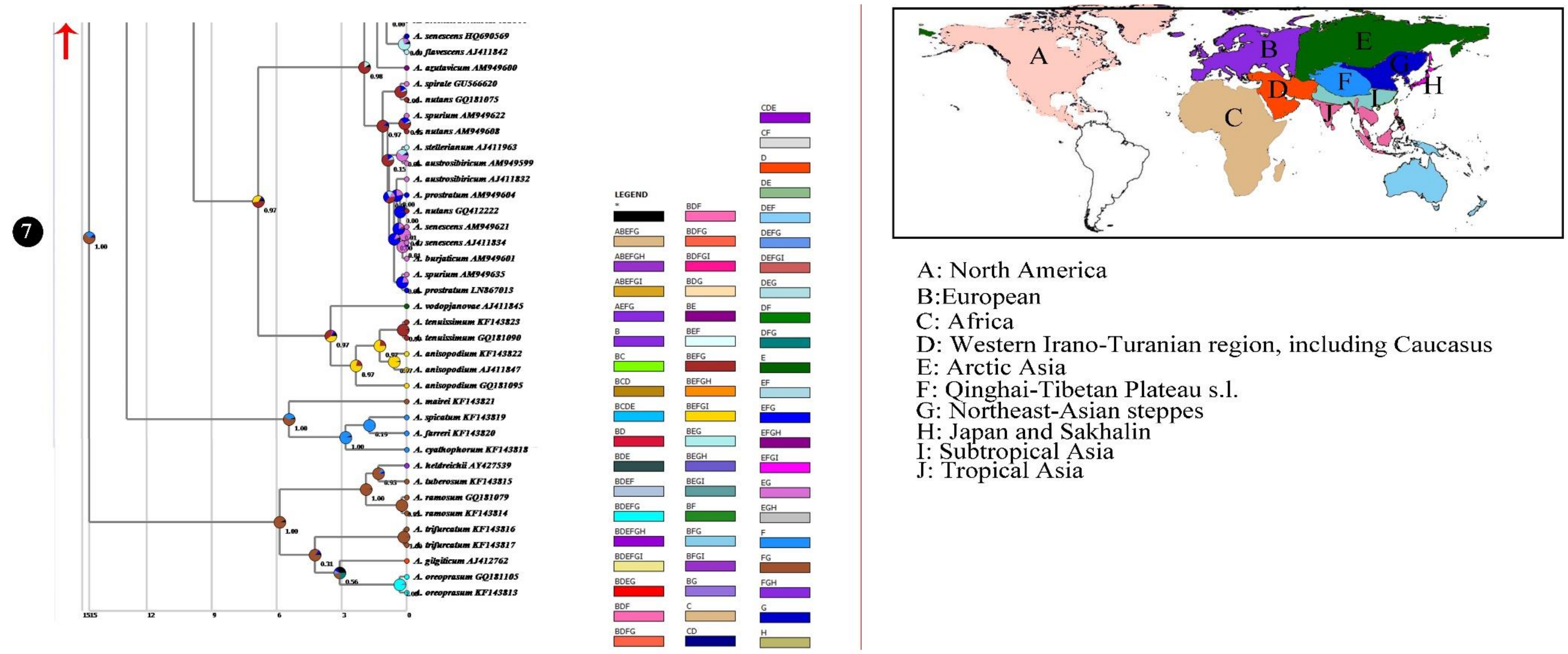

Fig. S8 RASP-based geographical distribution of the third evolutionary line (EL3) of the genus Allium based on the nrITS sequences.

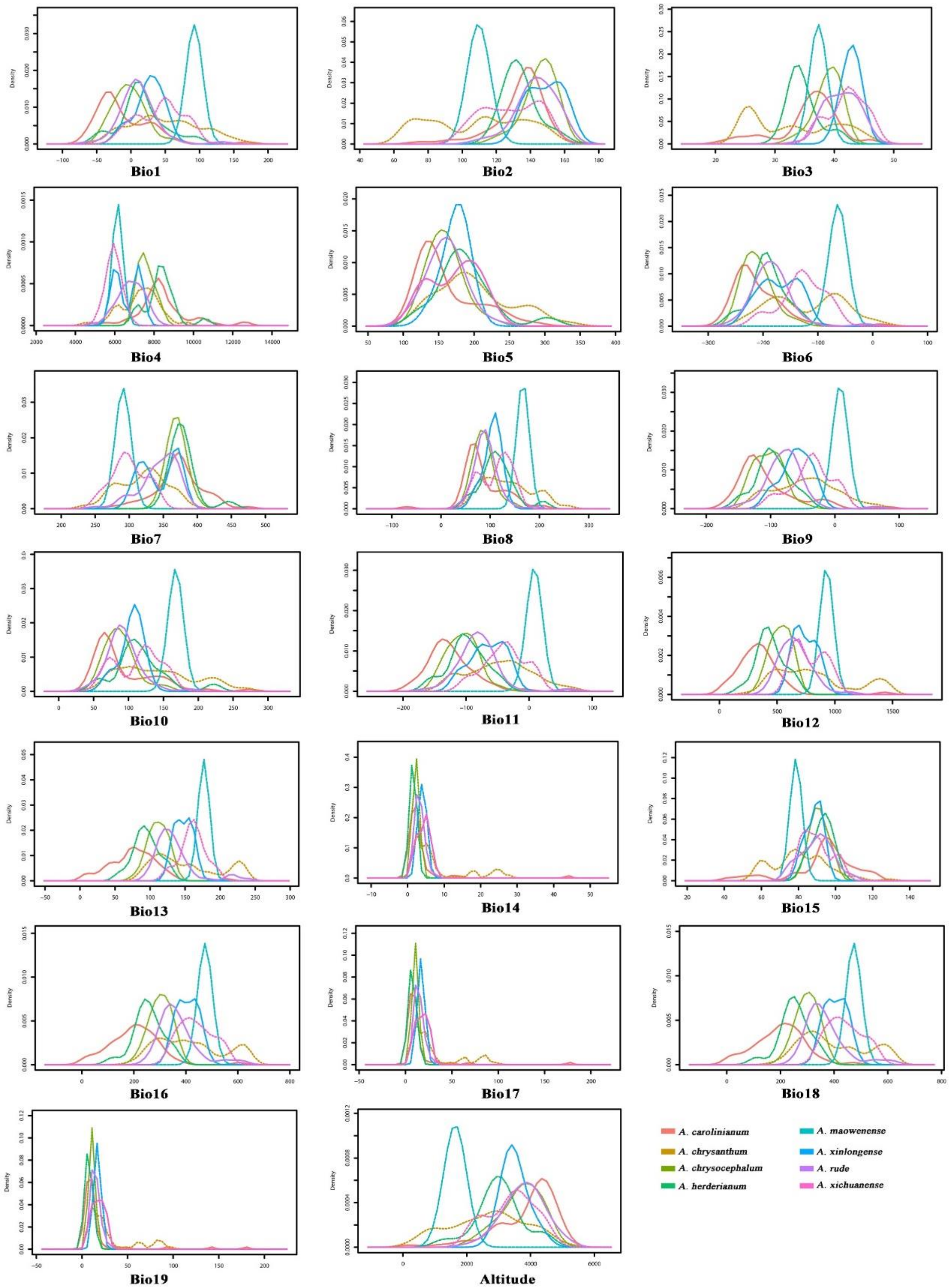

Fig. **S9 Density plots of the Asian sect. *Daghestanica* species for 20 bioclimatic variables.** Significance values of differences among species for each variable, tested using 10 000 bootstraps. In most density profiles, *A. carolinianum* have two peaks representing samples in the Qinghai and Tibet and XinJiang Province.

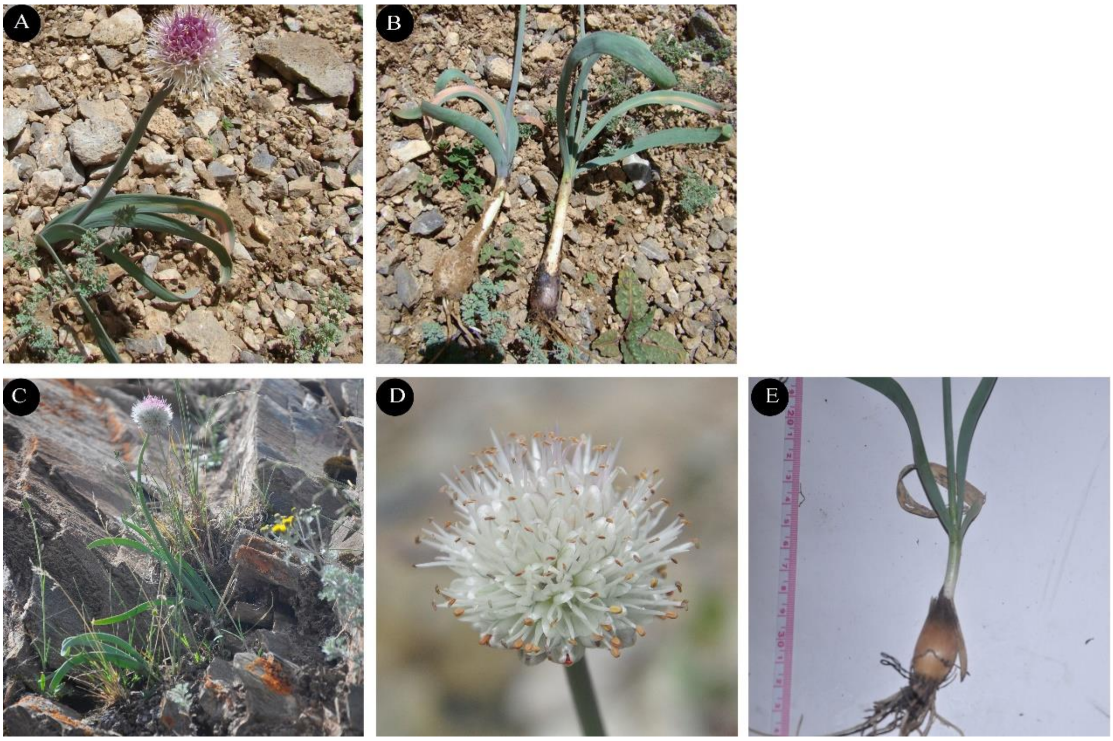

Fig. S10 Morphological comparisons among the populations of *A. carolinianum* collected in different locations. (A–B) Plants collected in Tibet, with the nrITS sequence number GQ181097. (C–E) Plants collected in Qinghai Province, coded with *A. carolinianum* 1–4.

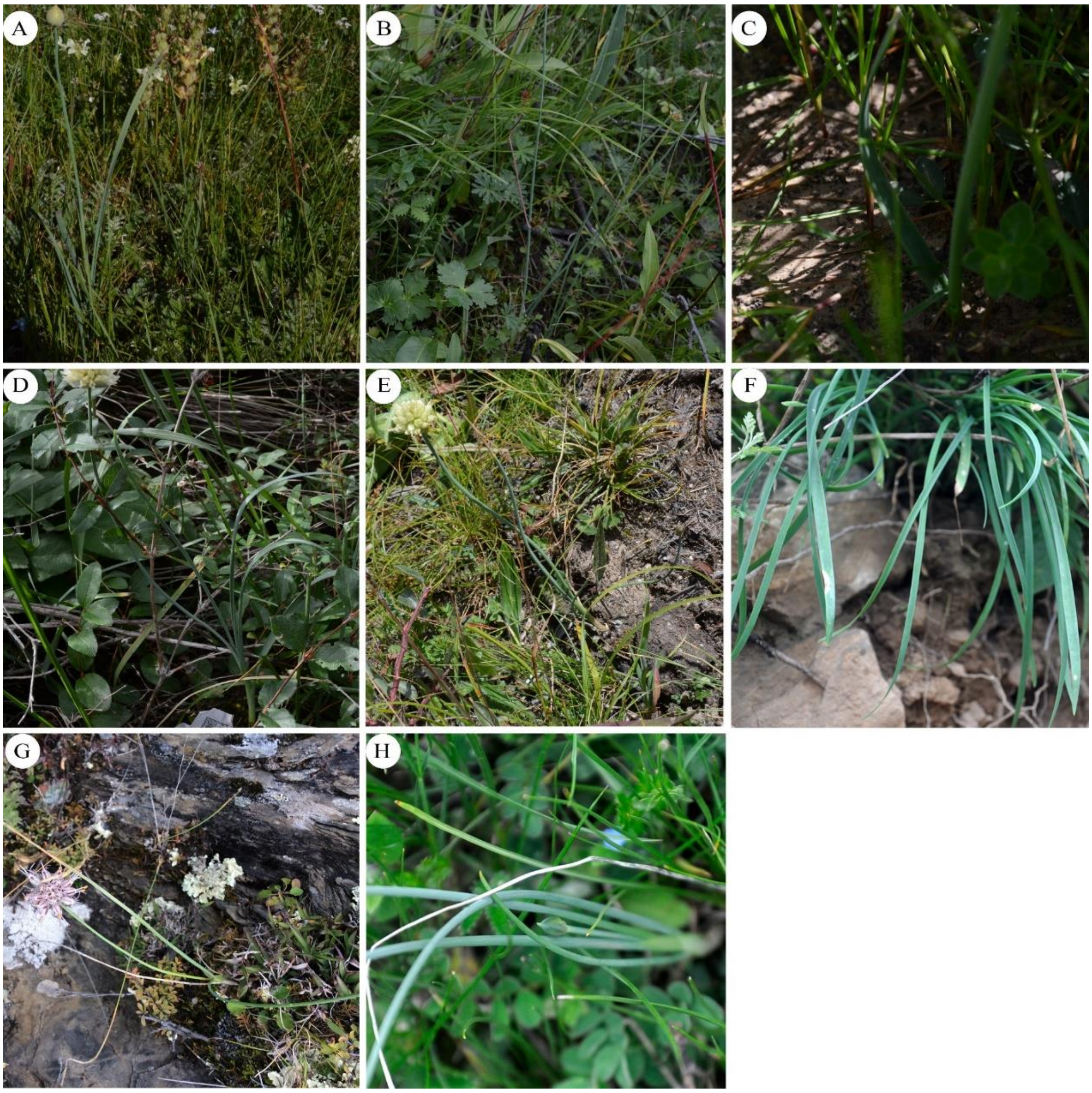

Fig. S11 Leaf comparisons among the seven Asian sect. *Daghestanica*. (A) *A. rude*; (B*) A. chrysanthum*; (C) *A. chrysocephalum*; (D) *A. herderianum*; (E) *A. xichuanense*; (F) *A. maowenense*; (G, H) *A. xinlongense*.

Table S1 The detailed information for the samples used in this study.

Species	**Location**	**Coordinates**	**Voucher**
A. herderianum	LianHuaShan, KangLe, GanSu	N34.934; E103.76	YHX2016081407
***A. xichuanense* 1**	MuGeCuo, KangDing, SiChuan	N30.14; E101.85	YHX2016082109
***A. xichuanense* 2**	DaPaoShan, KangDing, SiChuan	N30.28; E101.84	YHX2016082110
***A. xichuanense* 3**	SeDaXian, SiChuan	N32.27; E100.33	gxl20150921
***A. rude* 1**	BaLangShan, XiaoJinXian, SiChuan	N30.94; E102.90	YHX20160724
***A. rude* 2**	RuoErGai, RuoErGaiXian, SiChuan	N33.37; E102.45	YHX20160810
***A. rude* 3**	LaPuLengSi, XiaHeXian, GanSu	N35.19; E102.53	YHX20160812
***A. rude* 4**	BaiMaXueShan, DeQin, YunNan	N28.33; E99.08	YHX20160831
***A. rude* 5**	XueBaoDing, SongPanXian, SiChuan	N32.72; E103.74	YHX20160918
***A. rude* 6**	ShenXianChi, JiuZhaiGou, SiChuan	N33.49; E103.62	YHX20160919
***A. rude* 7**	NiMuTeXiang, HeNanXian, QingHai	N34.59; E101.34	LMJ2013071819
***A. rude* 8**	ZhaDuoXiang, ChengDuoXian, QingHai	N33.77; E96.74	LMJ2013072331
***A. rude* 9**	YuLong, JiangDaXian, Tibet	N31.50; E98.22	LMJ2013080251
***A. rude* 10**	QueErShan, DeGeXian, SiChuan	N31.94; E98.92	LMJ2013080461
***A. rude* 11**	LangMuSi, LuQuXian, GanSu	N34.09; E102.64	LMJ2013081474
***A. rude* 12**	WaiXiangSi, MaQuXian, GanSu	N34.94; E103.77	LMJ2013081576
***A. rude* 13**	DongDaShan, ZuoGongXian, Tibet	N29.43; E97.56	LMJ2013082986
***A. rude* 14**	RongXue, MangKangXian, Tibet	N29.42; E98.05	LMJ2013082988
***A. rude* 15**	LangBeiGou, YaJiangXian, SiChuan	N29.983; E100.90	LY2013092005
***A. rude* 16**	BaiYuXiang, JIuZhiXian, QingHai	N33.28; E100.67	LMJ2014092002
***A. rude* 17**	BanMaXian, QingHai	N32.93; E100.74	LMJ2014092108
***A. rude* 18**	JiangRang, MaQinXian, QingHai	N34.71; E100.24	LMJ2014092314
***A. rude* 19**	RangTangXian, SiChuan	N32.27; E100.98	H2010091402
***A. rude* 20**	SiTongXiang, DanBaXian, SiChuan	N30.79; E101.75	xqy2014092503-1
***A. rude* 21**	RangTangXIan, SiChuan	N32.27; E100.98	2010091401
***A. chrysocephalum* 1**	GaHai. LuQuXian, GanSu	N34.23; E102.23	YHX2016081103
***A. chrysocephalum* 2**	LaPuLengSi, XiaHeXian, GanSu	N35.16; E102.58	YHX2016081205

***A. chrysocephalum* 3**	ZuoGaiDuoMaXiang. HeZuoShi, GanSu	N35.07; E103.31	YHX2016081408
***A. chrysocephalum* 4**	ZhaDuoXiang, ChengDuoXian, QingHai	N33.77; E96.74	LMJ2013072434
***A. chrysocephalum* 5**	BaGanXiang, QuMaLaiXian, QingHai	N33.96; E96.56	LMJ2013072329
***A. chrysocephalum* 6**	ZhaDuoXiang, ChengDuoXian, QingHai	N33.77; E96.74	LMJ2013072332
***A. chrysanthum* 1**	ShuangChaXiang, LuQuXian, GanSu	N34.52; E102.77	YHX2016081104
***A. chrysanthum* 2**	QueErShan, DeGe, SiChuan	N31.94; E98.92	LMJ2013080463
***A. chrysanthum* 3**	ZongLaShan, BaTangXian, SiChuan	N29.56; E99.22	DYQ2014082401
***A. chrysanthum* 4**	SiGuNiangShan. RiLongZhen, SiChuan	N30.10; E102.85	T2011100903
***A. xinlongense* 1**	BanMaXian, QingHai	N32.93; E100.74	LMJ2013071206
***A. xinlongense* 2**	MaKeHe, BanMaXian, QingHai	N32.65; E100.98	LMJ2013071308
***A. xinlongense* 3**	BaiYuXiang, JiuZhiXian, QingHai	N33.28; E100.67	LMJ2014092003
***A. xinlongense* 4**	BanMaXian, QingHai	N32.93; E100.74	LMJ2014092107
***A. xinlongense* 5**	XinlongXian, QingHai	N31.21; E100.34	LMJ2014092112
***A. xinlongense* 6**	LiangHeXiang, XiaoJinXian, SiChuan	N31.48; E102.49	LMJ2013070239
***A. xinlongense* 7**	BaMei. DaoFuXian, SiChuan	N30.48; E101.48	T2011101147
***A. xinlongense* 8**	DaoFuXian, SiChuan	N30.48; E101.48	2015092641
***A. xinlongense* 9**	SuoMuXiang, MaErKangXian, SiChuan	N31.86; E102.48	2015091949
***A. xinlongense* 10**	DaoFuXian, SiChuan	N30.48; E101.48	LMJ2013080566
***A. maowenense* 1**	ShuiXiCun, MaoXian, SiChuan	N31.67; E103.83	LMJ2013062408
***A. maowenense* 2**	ShuiXiCun, MaoXian, SiChuan	N31.67; E103.83	LMJ2015082003
***A. carolinianum* 1**	JiangRang, MaQinXian, QingHai	N34.71; E100.24	LMJ2013071512
***A. carolinianum* 2**	AMingWaYangShan, XingHaiXian, QingHai	N35.59; E99.99	LMJ2013072024
***A. carolinianum* 3**	QiuZhiXiang, QuMaLai, QingHai	N34.13; E95.80	LMJ2013072237
***A. carolinianum* 4**	ZheDuoXiang, ChengDuoXian, QingHai	N33.77; E96.743	LMJ2013072330
Allium sp1	HeroBridge, HouXia, Urumqi, XinJiang	N43.36; E87.20	H11062603
Allium sp2	WulaShan, Baotou, Inner Mongolia	N40.70; E109.79	T2013080345
Allium sp3	Lanzhou, GanSu	N35.69;E104.16	LMJ2015091202
Allium sp4	TaiBaiXian, ShanXi	N34.00; E107.004	S11102601

*****_Allium_ species (_Allium_ sp1, sp2, sp3, sp4) were not determined were not used in the phylogenetic analyses.

Table S2 GenBank number newly produced in the study.

Species	nrITS	rps16	trnL-trnF	rpl32-trnL
Allium rude 1	MN866527	MN866582	MN866637	MN866692
Allium rude 2	MN866528	MN866583	MN866638	MN866693
Allium rude 3	MN866529	MN866584	MN866639	MN866694
Allium rude 4	MN866530	MN866585	MN866640	MN866695
Allium rude 5	MN866531	MN866586	MN866641	MN866696
Allium rude 6	MN866532	MN866587	MN866642	MN866697
Allium rude 7	MN866533	MN866588	MN866643	MN866698
Allium rude 8	MN866534	MN866589	MN866644	MN866699
Allium rude 9	MN866535	MN866590	MN866645	MN866700
Allium rude 10	MN866536	MN866591	MN866646	MN866701
Allium rude 11	MN866537	MN866592	MN866647	MN866702
Allium rude 12	MN866538	MN866593	MN866648	MN866703
Allium rude 13	MN866539	MN866594	MN866649	MN866704
Allium rude 14	MN866540	MN866595	MN866650	MN866705
Allium rude 15	MN866541	MN866596	MN866651	MN866706
Allium rude 16	MN866542	MN866597	MN866652	MN866707
Allium rude 17	MN866543	MN866598	MN866653	MN866708
Allium rude 18	MN866544	MN866599	MN866654	MN866709
Allium rude 19	MN866545	MN866600	MN866655	MN866710
Allium rude 20	MN866546	MN866601	MN866656	MN866711
Allium rude 21	MN866547	MN866602	MN866657	MN866712
Allium chrysocephalum 1	MN866548	MN866603	MN866658	MN866713
Allium chrysocephalum 2	MN866549	MN866604	MN866659	MN866714
Allium chrysocephalum 3	MN866550	MN866605	MN866660	MN866715
Allium chrysocephalum 4	MN866551	MN866606	MN866661	MN866716
Allium chrysocephalum 5	MN866552	MN866607	MN866662	MN866717
Allium chrysocephalum 6	MN866553	MN866608	MN866663	MN866718

Allium xichuanense 1	MN866554	MN866609	MN866664	MN866719
Allium xichuanense 2	MN866555	MN866610	MN866665	MN866720
Allium xichuanense 3	MN866556	MN866611	MN866666	MN866721
Allium chrysanthum 1	MN866557	MN866612	MN866667	MN866722
Allium chrysanthum 2	MN866558	MN866613	MN866668	MN866723
Allium chrysanthum 4	MN866559	MN866614	MN866669	MN866724
Allium chrysanthum 3	MN866560	MN866615	MN866670	MN866725
Allium herderianum	MN866561	MN866616	MN866671	MN866726
Allium maowenense 1	MN866562	MN866617	MN866672	MN866727
Allium maowenense 2	MN866563	MN866618	MN866673	MN866728
Allium xinlongense 6	MN866564	MN866619	MN866674	MN866729
Allium xinlongense 1	MN866565	MN866620	MN866675	MN866730
Allium xinlongense 2	MN866566	MN866621	MN866676	MN866731
Allium xinlongense 3	MN866567	MN866622	MN866677	MN866732
Allium xinlongense 4	MN866568	MN866623	MN866678	MN866733
Allium xinlongense 5	MN866569	MN866624	MN866679	MN866734
Allium xinlongense 7	MN866570	MN866625	MN866680	MN866735
Allium xinlongense 8	MN866571	MN866626	MN866681	MN866736
Allium xinlongense 9	MN866572	MN866627	MN866682	MN866737
Allium xinlongense 10	MN866573	MN866628	MN866683	MN866738
Allium carolinianum 1	MN866574	MN866629	MN866684	MN866739
Allium carolinianum 2	MN866575	MN866630	MN866685	MN866740
Allium carolinianum 3	MN866576	MN866631	MN866686	MN866741
Allium carolinianum 4	MN866577	MN866632	MN866687	MN866742
Allium sp1	MN866578	MN866633	MN866688	MN866743
Allium sp2	MN866579	MN866634	MN866689	MN866744
Allium sp3	MN866580	MN866635	MN866690	MN866745
Allium sp4	MN866581	MN866636	MN866691	MN866746

Table S3 GenBank number of the cpDNA downloaded in this study.

Species	trnL-trnF	Species	rpl32-trnL
Allium oreoprasum	KF143838	*Allium oreoprasum*	KF143871
Allium ramosum	KF143839	*Allium ramosum*	KF143872
Allium tuberosum	KF143840	*Allium tuberosum*	KF143873
Allium trifurcatum	KF143841	*Allium trifurcatum*	KF143874
Allium cyathophorum	KF143843	*Allium cyathophorum*	KF143876
Allium spicatum	KF143844	*Allium spicatum*	KF143877
Allium farreri	KF143845	*Allium farreri*	KF143878
Allium mairei	KF143846	*Allium mairei*	KF143879
Allium anisopodium	KF143847	*Allium anisopodium*	KF143880
Allium polyrhizum	KF143849	*Allium polyrhizum*	KF143882
Allium mongolicum	KF143851	*Allium mongolicum*	KF143884
Allium plurifoliatum	KF143854	*Allium plurifoliatum*	KF143887
Allium changduense	KF143855	*Allium changduense*	KF143888
Allium forrestii	KF143856	*Allium forrestii*	KF143889
Allium nutans	GU570988	*Allium nutans*	LN867033
Allium senescens	JF262654	*Allium senescens*	LN867039
Allium spirale	JF262656	*Allium spirale*	LN867040
Allium obliquum	GU570989	*Allium obliquum*	HG794143
Allium subtilissimum	KF143859	*Allium subtilissimum*	KF143892
Allium cepa	KC139046	*Allium cepa*	KC147465
Allium galanthum	KF143861	*Allium galanthum*	KF143894
Allium schoenoprasum	JF262662	*Allium schoenoprasum*	KF550149
Allium weschniakowii	KF143852	*Allium weschniakowii*	KF143885
Allium caespitosum	KF143857	*Allium caespitosum*	KF143890
Allium subtilissimum	KF143860	*Allium subtilissimum*	KF143893
Allium maximowiczii	JF262661	*Allium maximowiczii*	HE687220
Allium gunibicum_1	LR700293	*Allium gunibicum_1*	LR700250
Allium gunibicum_2	LR700294	*Allium gunibicum_2*	LR700251
Allium gunibicum_3	LR700295	*Allium gunibicum_3*	LR700252
Allium daghestanicum_2	LR700296	*Allium daghestanicum_2*	LR700253
Allium daghestanicum_3	LR700297	*Allium daghestanicum_3*	LR700254
Allium daghestanicum_1	LR700298	*Allium daghestanicum_1*	LR700255
Allium matinae	LR700299	*Allium matinae*	LR700256
Allium ericetorum_1	LR700300	*Allium ericetorum_1*	LR700257
Allium ericetorum_2	LR700301	*Allium ericetorum_2*	LR700258
Allium suaveolens_1	LR700302	*Allium suaveolens_1*	LR700259
Allium suaveolens_2	LR700303	*Allium suaveolens_2*	LR700260
Allium xichuanense	LR700304	*Allium xichuanense*	LR700261
Allium chrysocephalum	LR700305	*Allium chrysocephalum*	LR700262
Allium hymenorrhizum	LR700306	*Allium hymenorrhizum*	LR700263
Allium carolinianum	LR700307	*Allium carolinianum*	LR700264

Table S4 Bioclimatic variables extracted from World-Clim data set.

Variable	Description	Variable	Description
BIO1	Annual mean temperature	BIO11	Mean temperature of coldest quarter
BIO2	Mean diurnal range (mean of monthly (max temp – min temp))	BIO12	Annual precipitation
BIO3	Isothermality (BIO2/BIO7) (× 100)	BIO13	Precipitation of wettest month
BIO4	T ty (SD × 100)	BIO14	Precipitation of driest month
BIO5	Max temperature of warmest month	BIO15	Precipitation seasonality (coefficient of variation)
BIO6	Min temperature of coldest month	BIO16	Precipitation of wettest quarter
BIO7	Temperature Annual Range (BIO5–BIO6)	BIO17	Precipitation of driest quarter
BIO8	Mean temperature of wettest quarter	BIO18	Precipitation of warmest quarter
BIO9	Mean temperature of driest quarter	BIO19	Precipitation of coldest quarter
BIO10	Mean temperature of warmest quarter		

Table S5 The detailed information for 251 vetted localities of *A. carolianum* (CaR), *A. rude* (RuD), *A. chrysanthum* (ChR), *A. xichuanense* (XiC). *A. herderianum* (HeR), *A. chrysocephalum* (ChO), *A. maowenense* (MaO) and *A. xinlongense* (XiN).

Species	Latitude	Longitude (E)	Species	Latitude	Longitude (E)	Species	Latitude	Longitude (E)	Species	Latitude	Longitude (E)
CaR	102.77959	37.27708	RuD	102.5256	35.1999	ChR	102.347	36.92402	ChR	99.46135	27.20501
CaR	97.88839	36.48973	RuD	102.8296	34.56275	ChR	102.7631	37.21145	ChR	100.0067	28.25521
CaR	101.4184	37.46716	RuD	102.2257	34.63746	ChR	102.5964	37.44488	ChR	107.9905	33.52436
CaR	98.27184	37.82994	RuD	102.683	31.8494	ChR	102.8896	37.14321	ChR	103.5446	31.79166
CaR	102.15235	36.74861	RuD	102.3679	32.44261	ChR	95.62529	31.4075	ChR	106.5317	34.28041
CaR	96.73972	38.32451	RuD	100.8005	28.70882	ChR	108.3207	33.76936	ChR	101.8145	37.04542
CaR	94.3478	35.79299	RuD	103.8952	33.19839	ChR	109.1112	31.53027	ChR	108.9866	31.62383
CaR	87.61136	34.12212	RuD	104.0434	33.22429	ChR	108.9334	31.63594	ChR	99.07873	28.33382
CaR	84.76071	29.19306	RuD	104.1647	33.71252	ChR	102.1501	36.57758	ChR	86.54653	28.75238
CaR	79.69946	31.48331	RuD	95.61672	32.78343	ChR	110.2278	31.45695	XiC	102.3698	31.00617
CaR	81.28919	30.97159	RuD	100.2356	34.71161	ChR	102.4122	35.11185	XiC	102.6521	32.33537
CaR	80.09569	32.49151	RuD	95.52421	34.02789	ChR	100.109	29.1911	XiC	102.4804	30.38167
CaR	91.99585	31.50451	RuD	98.2533	33.07106	ChR	110.3188	31.39072	XiC	99.86653	28.82117
CaR	93.2459	31.28581	RuD	98.05938	32.92229	ChR	110.2694	31.58111	XiC	102.0071	30.52478
CaR	93.08361	35.21751	RuD	99.43025	33.72488	ChR	110.3665	31.43074	XiC	100.7621	30.17475
CaR	100.6901	36.9974	RuD	99.95567	33.86234	ChR	110.3008	31.43289	XiC	101.8015	30.2576
CaR	91.69469	32.27366	RuD	100.9038	33.15564	ChR	110.5071	31.49025	XiC	99.07873	28.33367
CaR	96.52597	33.89589	RuD	95.6224	32.7918	ChR	110.3972	31.38803	XiC	99.74066	31.6605
CaR	98.86059	35.10131	RuD	100.7286	31.8655	ChR	110.676	31.74502	HeR	102.1469	33.86099
CaR	95.58551	32.30829	RuD	101.8463	33.4481	ChR	110.3482	31.63944	HeR	101.3671	38.08352

CaR	94.46421	32.82454	RuD	103.3021	33.29889	ChR	94.05742	31.9185	HeR	102.633	34.09077
CaR	88.83691	33.18763	RuD	98.47312	31.63922	ChR	102.6719	31.84971	HeR	103.7558	35.00111
CaR	93.70262	30.39858	RuD	104.0266	33.53721	ChR	102.9619	33.576	HeR	103.0605	35.12452
CaR	97.9141	35.05206	RuD	101.1835	31.23273	ChR	107.2322	34.02623	HeR	102.0009	37.28584
CaR	95.13055	33.05927	RuD	99.43025	33.72488	ChR	109.0017	33.80752	HeR	99.82155	35.71752
CaR	98.13338	34.79742	RuD	100.109	29.1911	ChR	103.8485	31.68483	HeR	101.567	37.11098
CaR	97.91207	36.01003	RuD	102.4673	33.40983	ChR	102.4607	36.79184	HeR	101.6144	36.9701
CaR	94.31206	35.74396	RuD	104.6286	34.21018	ChR	102.4607	36.79198	HeR	101.2372	37.62177
CaR	96.69859	35.87229	RuD	100.2364	34.2071	ChR	95.28578	32.71977	HeR	100.7996	37.01133
CaR	95.62529	31.4075	RuD	101.3633	32.60423	ChR	106.2197	33.66035	HeR	100.8533	38.19986
CaR	97.36773	37.32021	RuD	98.99833	32.04868	ChR	97.25242	32.34923	HeR	100.942	38.21834
CaR	99.02373	36.69467	RuD	101.245	33.75423	ChR	103.2563	33.81356	HeR	100.1941	38.15125
CaR	96.69711	34.15211	RuD	102.5145	35.19627	ChR	102.7796	37.27722	HeR	101.9474	36.8458
CaR	96.77652	33.94634	RuD	99.64753	30.27392	ChR	107.276	36.59149	HeR	105.0733	37.58857
CaR	98.90495	35.18751	RuD	103.3534	34.50375	ChR	107.9015	34.09337	HeR	99.31276	35.38638
CaR	97.8968	35.05147	RuD	102.6012	32.23037	ChR	91.68258	32.265	ChO	98.10294	32.97848
CaR	99.8775	35.9668	RuD	102.5988	33.08605	ChR	100.9568	36.69105	ChO	102.7209	34.48879
CaR	98.08464	29.93553	RuD	100.2635	31.53855	ChR	108.626	34.11334	ChO	103.6625	34.9607
CaR	99.20087	35.34914	RuD	100.8005	28.70882	ChR	100.7973	37.12199	ChO	102.2257	34.63746
CaR	96.52557	33.89642	RuD	99.63452	33.7325	ChR	92.40191	32.11257	ChO	102.8296	34.56275
CaR	99.13426	33.8943	RuD	102.4474	32.33612	ChR	110.6745	30.19998	ChO	102.3675	34.25789
CaR	95.66068	34.56673	RuD	102.7725	26.80093	ChR	104.0351	33.00442	ChO	96.089	33.35135
CaR	99.51244	35.49647	RuD	97.77927	31.36016	ChR	102.5367	30.99955	ChO	99.95567	33.86234
CaR	95.49492	34.3037	RuD	98.92347	31.93998	ChR	110.3058	31.43084	ChO	101.389	34.32055
CaR	92.44732	34.21695	RuD	102.633	34.09077	ChR	102.2348	31.32246	ChO	100.7266	34.70507
CaR	111.71705	27.79181	RuD	102.0675	34.02389	ChR	107.9015	34.09337	ChO	98.85491	35.12971

CaR	98.13338	34.79742	RuD	97.97165	29.74736	ChR	103.1066	30.50271	ChO	96.22798	34.08697
CaR	90.00108	31.39854	RuD	98.17899	29.58197	ChR	103.5226	32.90068	ChO	101.1135	35.15283
CaR	87.19375	31.80661	RuD	103.8335	32.73756	ChR	106.251	33.70157	ChO	102.4382	36.91361
CaR	96.50914	37.65433	RuD	103.7741	33.30627	ChR	107.9015	34.09337	ChO	101.3197	34.34851
CaR	93.60162	35.43168	RuD	101.3365	34.59122	ChR	107.5203	33.80917	ChO	101.0737	34.38987
CaR	93.6726	33.89001	RuD	96.74369	33.76704	ChR	109.3038	29.79237	ChO	100.463	35.01551
CaR	87.93931	43.5404	RuD	100.7369	32.93262	ChR	103.537	35.13771	ChO	100.9346	33.52821
CaR	77.14555	37.37014	RuD	100.9707	32.2852	ChR	102.3679	32.44276	ChO	97.10648	33.37324
CaR	87.27031	43.46963	RuD	100.2068	31.75027	ChR	103.5905	31.8976	ChO	97.34782	33.13057
CaR	84.54515	43.26706	RuD	102.9012	30.93465	ChR	104.314	34.42339	ChO	99.94586	34.47718
CaR	75.23102	37.78518	ChR	108.843	32.05391	ChR	102.6719	31.84971	ChO	96.77652	33.94678
CaR	88.37208	37.09018	ChR	108.4289	31.20373	ChR	102.6484	30.94229	ChO	101.5499	34.34989
CaR	74.85987	37.00736	ChR	104.9206	34.42519	ChR	102.7951	31.58543	ChO	102.9373	35.30153
CaR	73.96635	39.71185	ChR	99.19021	34.47662	ChR	104.093	34.9628	MaO	103.826	31.66998
CaR	76.9839	36.83613	ChR	102.1989	37.01405	ChR	100.1678	26.84245	MaO	103.5114	31.38434
